# Synthesis of Piperine-Based Ester Derivatives with Diverse Aromatic Rings and Their Agricultural Bioactivities against *Tetranychus cinnabarinus* Boisduval, *Aphis citricola* Van der Goot, and *Eriosoma lanigerum* Hausmann

**DOI:** 10.3390/insects14010040

**Published:** 2022-12-31

**Authors:** Tianze Li, Min Lv, Houpeng Wen, Yanyan Wang, Sunita Thapa, Shaoyong Zhang, Hui Xu

**Affiliations:** 1 College of Plant Protection, Northwest A&F University, Xianyang 712100, China; 2 Key Laboratory of Vector Biology and Pathogen Control of Zhejiang Province, College of Life Science, Huzhou University, Huzhou 313000, China

**Keywords:** piperine, ester, *Tetranychus cinnabarinus* Boisduval, *Aphis citricola* Van der Goot, *Eriosoma lanigerum* Hausmann, structural modification, insecticidal activities, SEM, VHA reaction

## Abstract

**Simple Summary:**

Piperine, as a plant-derived natural product, has diverse bioactivities in the agricultural industry. Although the chemical insecticides/acaricides are now used to control pests, the increasingly serious resistance has raised an arduous challenge for their effective pest management. In this paper, by using piperine as a lead compound, a series of its novel ester derivatives were obtained by structural modification with different aromatic rings. Evaluation of the activities was conducted against three crop-threatening pests, *Tetranychus cinnabarinus* Boisduval (Acari: Tetranychidae), *Aphis citricola* Van der Goot (Homoptera: Aphididae), and *Eriosoma lanigerum* Hausmann (Hemiptera: Aphididae). Some compounds exhibited good to excellent acaricidal and aphicidal activities. These results further indicate that the naturally occurring compound piperine can be used as a lead compound to develop potential agrochemicals for pest management.

**Abstract:**

Exploration of plant secondary metabolites or by using them as leads for development of new pesticides has become one of the focal research topics nowadays. Herein, a series of new ester derivatives of piperine were prepared via the Vilsmeier–Haack–Arnold (VHA) reaction, and their structures were characterized by infrared spectroscopy (IR), melting point (mp), proton nuclear magnetic resonance spectroscopy (^1^H NMR), and carbon nuclear magnetic resonance spectroscopy (^13^C NMR). Notably, the steric configurations of compounds **6** and **7** were confirmed by single-crystal analysis. Against *T. cinnabarinus*, compounds **9** and **11** exhibited 47.6- and 45.4-fold more pronounced acaricidal activity than piperine. In particular, compounds **9** and **11** also showed 2.6-fold control efficiency on the fifth day of piperine. In addition, compound **6** (>10–fold higher than piperine) displayed the most potent aphicidal activity against *A. citricola*. Furthermore, some derivatives showed good aphicidal activities against *E. lanigerum*. Moreover, the effects of compounds on the cuticles of *T. cinnabarinus* were investigated by the scanning electron microscope (SEM) imaging method. This study will pave the way for future high value added application of piperine and its derivatives as botanical pesticides.

## 1. Introduction

In the 21st century, new technologies have actively impacted many aspects of human life, including health management as well as food quality and safety. However, because of the rapid growth of the population and the expansion of urbanization leading to a decrease in the area of agricultural acreage, it is hard to produce enough food for everyone in the world. Thus, scientific research should focus on deficient food production and improve the yield and quality. Nevertheless, the limitation is that pests on crops cannot be controlled effectively [1,2]. The carmine spider mite, *Tetranychus cinnabarinus* Boisduval (Acari: Tetranychidae), is one of the most dominating agricultural pests, which can cause major destruction for more than 100 species of plants [3,4]. The aphids, *Aphis citricola* Van der Goot (Homoptera: Aphididae) and *Eriosoma lanigerum* Hausmann (Hemiptera: Aphididae), can spread plant viruses on a large scale, generate an infection of fungi, and affect the process of photosynthesis by their hazardous way of feeding [5,6]. For example, because of their specific mouthpart structure, the Hemiptera pests can act as vectors for most plant viruses, such as *Barley yellow dwarf virus* (BYDV), *Potato leafroll virus* (PLRV), *Turnip yellows virus* (TuYV), *Cereal yellow dwarf virus* (CYDV), and *Pea enation mosaic virus 1* (PEMV1) [7,8]. The abovementioned acarids and aphids have become the most destructive pests in agriculture production and cause hundreds of millions of USD in economic losses annually [9]. In addition, the unconscionable abuse of chemical industrial pesticides has aroused increasingly serious resistance, not only because of environmental pollution but also toxicities to natural enemies [10,11,12]. Therefore, the development of sustainable and eco-friendly pesticides is extremely urgent. Fortunately, plant-derived natural products have been used to discover green pesticidal molecules, which could delay the development of resistance and mitigate the pollution of synthetic insecticides/acaricides/aphicides [13]. 

**Figure 1:**
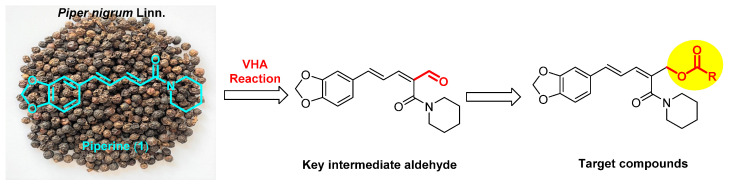
Design of target compounds from piperine (**1**) via the VHA reaction.

Piperine (1, Figure 1) is an alkaloid isolated from *Piper nigrum* Linn. This natural compound has attracted attention because of its numerous bioactivities including anti-cancer [14,15,16], anti-inflammation [17], anti-Alzheimer’s disease [18], anti-malarial [19], anti-diabetic [20], anti-oxidant [21], and anti-mycobacterial activities [22]. Moreover, piperine also has agricultural benefits such as anti-fungal [23] and insecticidal activities [24,25,26,27]. On the other hand, the ester group introduced on natural products could result in potent derivatives possessing insecticidal [28,29,30,31,32,33], anti-fungal [34,35], and herbicidal activities [36]. Recently, the Vilsmeier–Haack–Arnold (VHA) reaction has become a general method for the formylation of various active aromatic hydrocarbons [37]. In this paper, a series of new piperine ester derivatives with diverse aromatic rings were designed and synthesized by application of the VHA reaction (Figure 1). Their bioactivities were evaluated against *T. cinnabarinus*, *A. citricola*, and *E. lanigerum*. Additionally, the scanning electron microscope (SEM) imaging method was used to investigate the effects of piperine ester derivatives on the cuticle of *T. cinnabarinus*.

## 2. Materials and Methods

### 2.1. Insects

The spider mites (*T. cinnabarinus*) were reared by using cowpea seedlings as the material; under the conditions of no acaricides, 26 ± 1 °C, 70–75% RH (relative humidity), and 14 h/10 h (light/dark) photoperiod in the laboratory. The aphids (*A. citricola*, and *E. lanigerum*) were collected from apple trees in experimental fields at the Plant Protection College of the Northwest A&F University (Yangling, Shaanxi province, China).

### 2.2. Reagents and Instruments

All chemical reagents were purchased and utilized without further purification. Solvents were used directly or treated with standard methods before use. Analytical thin-layer chromatography (TLC) and preparative thin-layer chromatography (PTLC) were performed with silica gel plates using silica gel 60 GF_254_ (Qingdao Haiyang Chemical Co., Ltd., Qingdao, China). Silica-gel column chromatography was performed with silica gel 200–300 mesh (Qingdao Haiyang Chemical Co., Ltd., Qingdao, China). The melting point (mp) was determined using the XT-415 digital melting point apparatus (Beijing Tech Instrument, Ltd., Beijing, China) and was uncorrected. Nuclear magnetic resonance spectra (^1^H NMR, ^13^C NMR) were obtained using Avance III 400/500 MHz equipment (Bruker, Karlsruhe, Germany) and using tetramethylsilane (TMS) as the internal standard. X-ray crystallography was performed on SMART APEX II equipment (Bruker, Karlsruhe, Germany). Infrared (IR) spectra were obtained from a TENSOR27 instrument (Bruker, Karlsruhe, Germany). The scanning electron microscope images were shot using an S-3400N scanning electron microscope (Hitachi, Tokyo, Japan). Piperine (**1**) was purchased from Baoji Haoxiang Biotechnology Co. Ltd. (Shaanxi province, China).

### 2.3. General Procedure for the Synthesis of Aldehyde 2

To a stirred solution of phosphorus oxychloride (POCl_3_, 20 mmol) in N,N-dimethylformamide (DMF, 20 mmol) at 0 °C for 0.5 h, a solution of piperine (**1**, 4 mmol) in DMF (2 mL) was added dropwise. After the addition, the mixture was allowed to stir at 60 °C. When the reaction was complete and checked by TLC analysis, the mixture was poured into ice water (200 mL). Subsequently, 1 M aq. NaOH solution was used to neutralize the pH value to 8–9 and the precipitated product was collected and washed with ice water and MeOH to afford aldehyde **2**.

*Data for **2*****:** Yield: 30%; yellow solid; mp 184–186 °C; IR cm^–1^ (KBr): 3052, 2937, 2858, 2733, 1678, 1607, 1445, 1256, 1188, 812, 707; ^1^H NMR (500 MHz, CDCl_3_) *δ*: 9.49 (s, 1H, –C*H*O), 7.15 (d, *J* = 11.0 Hz, 1H), 6.97–7.03 (m, 3H), 6.80–6.89 (m, 2H), 6.01 (s, 2H, –OC*H*_2_O–), 3.74 (s, 2H, –NC*H*_2_–), 3.26 (t, *J* = 6.0 Hz, 2H, –N*CH_2_*–)*,* 1.66 (s, 4H), 1.54 (s, 2H); ^13^C NMR (125 MHz, CDCl_3_) *δ*: 189.7, 163.8, 149.6, 149.4, 148.4, 144.5, 137.4, 129.9, 124.4, 121.5, 108.6, 106.3, 101.6, 47.9, 42.5, 26.6, 25.8, 24.4. HRMS [ESI]: calcd for C_18_H_19_NO_4_Na ([M + Na]+), 336.1206; found, 336.1206.

### 2.4. General Procedure for the Synthesis of Compound **3**

A mixture of aldehyde **2** (1 mmol) and sodium borohydride (NaBH_4_, 1.5 mmol) in MeOH (5 mL) was stirred at 0 °C for 0.5 h. Then, it was quenched using 0.2 M aq. HCl solution and extracted with ethyl acetate (EtOAc, 20 mL × 3). Then, the combined organic phase was washed with brine (30 mL × 3), dried over anhydrous Na_2_SO_4_, concentrated in vacuo, and purified by silica-gel column chromatography to give compound **3**.

*Data for****3***: Yield: 99%; yellow solid; mp 123–125 °C; IR cm^–1^ (KBr): 3270, 2939, 2866, 1618, 1441, 1249, 1190, 811, 719; ^1^H NMR (500 MHz, CDCl_3_) *δ*: 6.87 (d, *J* = 0.5 Hz, 1H), 6.77–6.79 (m, 1H), 6.74 (d, *J* = 8.0 Hz, 1H), 6.48–6.55 (m, 2H), 6.32–6.34 (m, 1H), 5.95 (s, 2H, –OCH_2_O–), 4.30 (s, 2H, –CH_2_OH), 3.67–3.69 (m, 2H, –NCH_2_–), 3.45 (t, *J* = 5.5 Hz, 2H, –NCH_2_–), 1.63–1.64 (m, 4H), 1.52 (t, *J* = 5.5 Hz, 2H); ^13^C NMR (125 MHz, CDCl_3_) *δ*: 168.8, 148.1, 147.6, 136.8, 134.9, 131.2, 127.4, 122.2, 121.8, 108.4, 105.5, 101.2, 63.9, 47.8, 42.4, 26.6, 25.8, 24.4. HRMS [ESI]: calcd for C_18_H_21_NO_4_Na ([M + Na]^+^), 338.1363; found, 338.1354.

### 2.5. General Procedure for the Synthesis of Target Compounds **4**–**12**

A mixture of compound **3** (0.2 mmol), aromatic carboxylic acids (RCO_2_H, 0.4 mmol), 1-(3-dimethylaminopropyl)-3-ethylcarbodiimide hydrochloride (EDCI, 0.4 mmol), and 4-dimethylaminopyridine (DMAP, 0.04 mmol) in dry dichloromethane (CH_2_Cl_2_, 5 mL) was stirred at 0 °C to room temperature. When the reaction was complete the solution was checked by TLC analysis and the mixture was concentrated and purified by PTLC to afford compounds **4–12** in 44–57% yields. The characterization data for **4** and **5** are presented here, whereas **6–12** are characterized in the Supporting Information.

*Data for **4***: Yield: 53%; yellow solid; mp 106–107 °C; IR cm^–1^ (KBr): 3429, 3051, 2932, 2353, 1723, 1588, 1484, 1447, 1241, 1183, 1029, 958, 764, 598; ^1^H NMR (400 MHz, CDCl_3_) *δ*: 7.58 (d, *J* = 0.8 Hz, 1H), 7.19 (d, *J* = 2.8 Hz, 1H), 6.90 (d, *J* = 1.2 Hz, 1H), 6.82–6.84 (m, 1H), 6.77 (d, *J* = 8.0 Hz, 1H), 6.51–6.60 (m, 3H), 6.46 (d, *J* = 10.4 Hz, 1H), 5.97 (s, 2H), 5.04 (s, 2H), 3.71 (s, 2H), 3.46 (t, *J* = 5.2 Hz, 2H), 1.64 (s, 4H), 1.48 (s, 2H); ^13^C NMR (100 MHz, CDCl_3_) δ: 167.3, 158.2, 148.2, 148.0, 146.5, 144.3, 136.7, 131.7, 130.9, 130.5, 122.2, 121.8, 118.2, 111.9, 108.5, 105.5, 101.2, 66.5, 47.8, 42.5, 26.5, 25.8, 24.5.

*Data for **5***: Yield: 53%; yellow oil; IR cm^–1^ (KBr): 3399, 3307, 3075, 2373, 1723, 1579, 1491, 1450, 1368, 1284, 1121, 1026, 931, 741, 701, 605; ^1^H NMR (400 MHz, CDCl_3_) *δ*: 9.21 (d, *J* = 1.6 Hz, 1H), 8.79 (dd, *J_1_* = 4.8 Hz, *J_2_* = 1.6 Hz, 1H), 8.32 (dt, *J_1_* = 8.0 Hz, *J_2_* = 2.0 Hz, 1H), 7.43 (dd, *J_1_* = 8.0 Hz, *J_2_* = 5.2 Hz, 1H), 6.91 (d, *J* = 1.2 Hz, 1H), 6.83–6.85 (m, 1H), 6.78 (d, *J* = 8.0 Hz, 1H), 6.53–6.66 (m, 2H), 6.50 (d, *J* = 10.4 Hz, 1H), 5.97 (s, 2H), 5.11 (s, 2H), 3.72 (s, 2H), 3.47 (t, *J* = 5.2 Hz, 1H), 1.64 (s, 4H), 1.46 (s, 2H); ^13^C NMR (100 MHz, CDCl_3_) δ: 167.2, 164.8, 153.5, 150.7, 148.2, 148.1, 137.2, 137.0, 132.0, 130.7, 130.2, 125.9, 123.4, 122.3, 121.7, 108.5, 105.5, 101.3, 67.2, 47.7, 42.4, 26.6, 25.8, 24.4.

### 2.6. Biological Assay

#### 2.6.1. Acaricidal Activity against *T. cinnabarinus*

The acaricidal activities of compounds **1–12** against *T. cinnabarinus* were tested by using the slide-dipping method [38,39]. The commercial acaricidal pesticide spirodiclofen was used as a positive control. The solutions of spirodiclofen and piperine and its derivatives were prepared in aq. Tween-80 (0.1 g/L) at 0.5 mg/mL. A total of 35–45 healthy, active, and size-uniform female adults of *T. cinnabarinus* were selected and affixed dorsally in two lines to a strip of double-coated masking tape on a microscope slide (triplicate for each compound). Then the slides were dipped into the corresponding solution for 5 s and taken out. Additionally, the slides treated with 0.1 g/L of aq.Tween-80 were used as a blank control group (CK). Thereafter, these slides with mites were moisturized and kept at 26 ± 1 °C and 70–75% RH (relative humidity) with a 14 h/10 h (light/dark) photoperiod. The results were checked by a binocular dissecting microscope and the number of dead mites was recorded at 48 and 72 h after treatment. Finally, the corrected mortality rate was calculated as the formula:

$$\mathrm{Corrected}\;\mathrm{mortality}\;\mathrm{rate}\;(\%)=\frac{\left(T\;–\;C\right)}{\left(100\%\;–\;C\right)}\;100$$

where *C* is the mortality rate of CK and *T* is the mortality rate of the treated *T. cinnabarinus*. Furthermore, linear regressions of the 72 h mortality rates (%) versus five concentrations of some potent compounds and spirodiclofen were performed and their LC_50_ values were calculated.

#### 2.6.2. Control Efficiency of Some Compounds against *T. cinnabarinus* in the Greenhouse

The solutions of compounds **1**, **9**, **11**, and spirodiclofen were prepared in aq. Tween-80 (1 g/L) at 0.3 mg/mL. Each cowpea seedling (three seedlings per replicate and three replicates per treatment) was infected with 50 healthy and size-uniform female adults of *T. cinnabarinus* in the greenhouse. An airbrush was used to spray 10 mL of the corresponding solution for each treatment. The blank control group was tested with aq. Tween-80 (1 g/L) only. The experimental conditions and evaluation method were as mentioned above [38,39].

#### 2.6.3. Aphicidal Activity against *A. citricola*

The aphicidal activity against *A. citricola* was assessed by the topical application method. The solutions of compounds **1–12** were prepared at 1 mg/mL in acetone (CK was only treated with acetone), and methomyl (0.1 mg/mL) was used as a positive control. Then, approximately 90 apterous adult aphids were selected for each compound (30 aphids per dish). Subsequently, 0.04 µL corresponding solution was dropped on the pronotum of each aphid. The experiment was carried out at 25 ± 1 °C, 50 ± 7% RH, and on a 14 h/10 h (light/dark) photoperiod. Their 24 and 48 h corrected mortality rate values were calculated as above. Finally, the 48 h LD_50_ values of some potent compounds were calculated [40,41].

#### 2.6.4. Aphicidal Activity against *E. lanigerum*

The solutions of compounds **1–12** were prepared at 0.2 mg/mL in 0.1 g/L aq. Tween-80 (0.1 g/L aq. Tween-80 as CK). Two commercial pesticides, thiamethoxam and chlorpyrifos, were used as positive controls. Some 2-year-old apple twigs were cut into pieces of 3–5 cm in length with one or two aphid groups (about 60 aphids) on each twig. Then, the 2 mL corresponding solution was sprayed on the twigs with an average of three replicates and the twigs were moisturized with cotton on both ends and incubated at 25 ± 1 °C, 60–80% RH, and with a 14 h/10 h (light/dark) photoperiod. The number of dead aphids was recorded at 48 and 72 h after treatment and the corrected mortality rate values were calculated as mentioned above [42].

#### 2.6.5. The Effects of Piperine Derivatives **9** and **11** on the Features of Mite Cuticles

After the mites were treated with compounds **9** (at 0.298 mg/mL) and **11** (at 0.313 mg/mL) for 24, 48, and 72 h and the corresponding mites were collected (0.1 g/L aq. Tween-80 as CK). They were treated with 4% aq. glutaraldehyde at 4 °C for 6 h, then the samples were washed 3 times with 0.1 M aq. phosphate-buffered saline (PBS) and a series of ethanol solutions (10%, 30%, 50%, 70%, 80%, 90%, 95%, and absolute ethanol). Subsequently, a series of *tert*–butanol solutions (25%, 50%, 75%, and 100% *tert*–butanol, *v/v* = ethanol/*tert*–butanol) were used to rinse the mites. After the samples were dried and gold sprayed, they were observed by SEM [43].

### 2.7. Statistical Analysis

All the recorded data were statistically analyzed using the software of IBM SPSS Statistics 20.0. The probit analysis was used to estimate the LC_50_ and LD_50_ values. The mortality rates were subjected to one-way ANOVA and Duncan’s test (*p* < 0.05).

## 3. Results and Discussion

### 3.1. Synthesis

**Figure 2:**
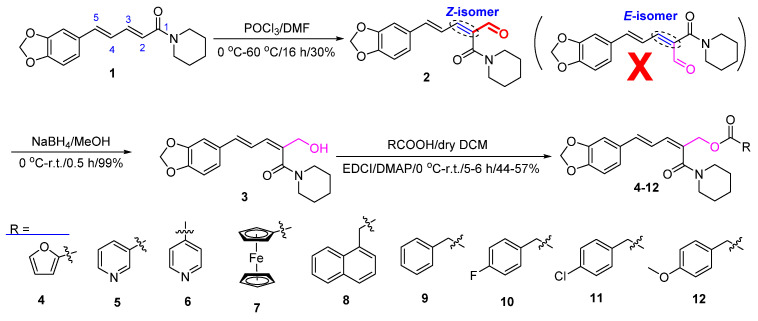
Synthetic route for the preparation of piperine ester derivatives (**2–12**).

**Figure 3:**
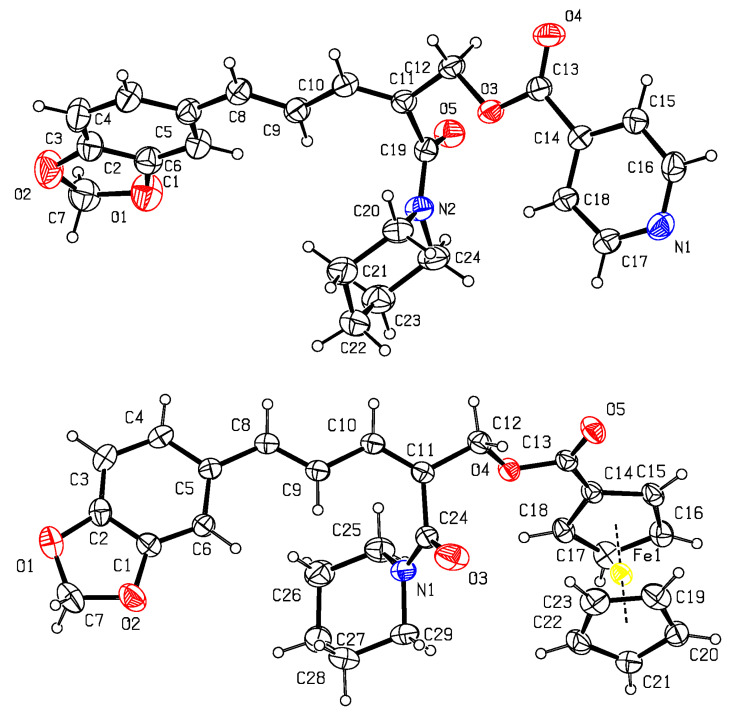
X–ray crystal structures of **6** (**top**) and **7** (**bottom**).

As shown in Figure 2, the formyl group was firstly introduced at the C–2 position of piperine (**1**) by the VHA reaction to afford the aldehyde **2** [44]. Then, in the presence of NaBH_4_ and MeOH, compound **2** was reduced to give compound **3** [45]. Finally, in the presence of EDCI and DMAP, target compounds **4–12** were obtained by reaction of compound **3** with aromatic acids RCO_2_H in 44–57% yields [46,47]. The chemical structures of compounds **2–12** were all determined by mp, IR, ^1^H NMR, and ^13^C NMR. Fortunately, the steric configurations of compounds **6** (CCDC: 2215963) and **7** (CCDC: 2215965) were confirmed by single-crystal X-ray diffraction (Figure 3). Obviously, the amide groups of compounds **6** and **7** were all in *Z* configuration. That is, the formylation reaction of compound **1** (its amide group in *E* configuration) with POCl_3_ and DMF was at its C-2 position and the amide group of compound **2** was in *Z* configuration. When compound **1** reacted with POCl_3_ and DMF, if the amide group of compound **1** was in *Z* configuration, there was possibly a larger space at the C-2 position for the VHA reaction.

### 3.2. Pesticidal Activities

#### 3.2.1. Acaricidal Activity against *T. cinnabarinus*

**Figure 4:**
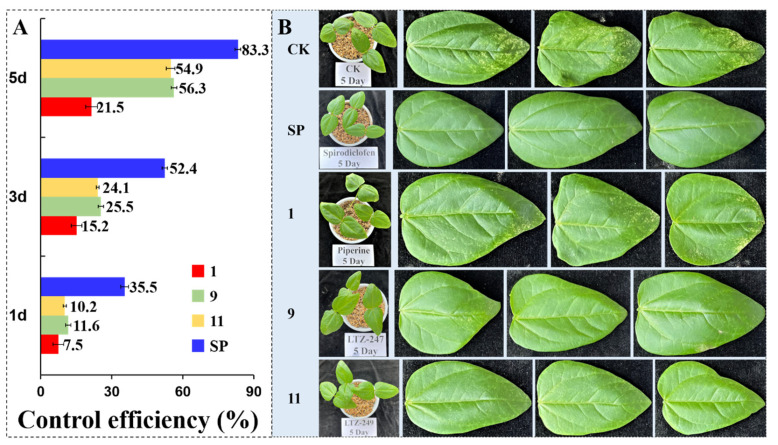
The control efficiency (**A**) and symptoms (**B**) of cowpea leaves infected with *T. cinnabarinus* on the 5th day after treatment with SP (spirodiclofen), **1** (piperine), **9** (LTZ–247), and **11** (LTZ–249) (CK: aq. Tween–80 (1 g/L)).

The 48 and 72 h acaricidal results of compounds **1–12** at 0.5 mg/mL against the female adults of *T. cinnabarinus* are described in Table 1. The 72 h corrected mortality rates (CMRs) of all derivatives against *T. cinnabarinus* ranged from 30.4% to 59.4%. In particular, the 72 h CMRs of compounds **9** (R as PhCH_2_) and **11** (R as *p*–ClPhCH_2_) were 59.4% and 56.3%, respectively; whereas the 72 h CMR of compound **1** was only 25.2%. Compounds **9** and **11** exhibited significant toxicities. It was revealed that R as benzyl or *p*–Clbenzyl was necessary for improving the acaricidal activity. In addition, the LC_50_ values of compounds **9** and **11** against *T. cinnabarinus* were 0.298 and 0.313 mg/mL, respectively (Table 2). Nevertheless, the LC_50_ value of compound **1** was 14.198 mg/mL. Particularly, compounds **9** and **11** were 45.4– and 47.6–fold stronger than piperine. Notably, their acaricidal activities were more potent than those of 7-oxycarbonylandrographolide, cholesterol, and osthole ester/amide derivatives [38,41,45,46]. When compared with our previous results [48], it suggested that introduction of the CH_2_ between the phenyl/4-Fphenyl/4-Clphenyl and the carbonyl groups was very important for the acaricidal activity. Later, to obtain good acaricidal agents, R as the different substituted benzyl should be considered. The cowpea seedling leaves turned from green to light yellow and the symptoms of densely packed white spots (sucked by *T. cinnabarinus*) were presented to the piperine-treated and the blank control groups (Figure 4). However, the leaves of the **9**- and **11**-treated groups appeared with sporadic white spots. These phenomena were the same as our previous reports [47]. The control effects on the fifth day of compounds **9** and **11** were 56.3% and 54.9%, respectively, which were 2.6-fold that of **1** (21.5%). The photographs for evaluation of the control effects of compounds **1**, **9**, **11**, and spirodiclofens are shown in the supporting information (Figures S1–S5).

#### 3.2.2. Aphicidal Activity against *A. citricola*

The 24 and 48 h aphicidal results of compounds **1–12** against apterous adults of *A. citricola* at 0.04 μg/nymph are depicted in Table 3. The CMRs at 48 h of compounds **2** (R as CHO) and **3** (R as CH_2_OH) were 18.2% and 27.3%, respectively, whereas the CMR at 48 h of compound **1** was 25.0%. It suggested that introduction of the formyl or hydroxymethyl group at the C-2 position of compound **1** was unnecessary for improving the aphicidal activity. However, introduction of the ester group at the C-2 position of compound **1** could lead to potent derivatives. For example, CMRs at 48 h of compounds **4–12** were 28.4%–55.7%, in particular compound **6** (R as pyridin-4-yl) showed the most potent aphicidal activity with a CMR at 48 h of 55.7%. The CMRs at 48 h of compounds **4** (R as furan-2-yl) and **5** (R as pyridin-3-yl) were only 29.5% and 28.4%, respectively. It indicated that introduction of the pyridin-4-ylcarbonyloxymethylene group at the C-2 position of compound **1** was very important for the aphicidal activity against *A. citricola*. Furthermore, the LD_50_ value of compound **6** was 0.030 µg/nymph, that is, the aphicidal activity of compound **6** was >10–fold of that of compound **1** (LD_50_: 0.308 µg/nymph) (Table 4). To our delight, the aphicidal activity of compound **6** was as promising as those of 7-oxycarbonylandrographolide and osthole ester/amide derivatives [38,46].

#### 3.2.3. Aphicidal Activity against *E. lanigerum*

The results of the aphicidal activity at 48 and 72 h of compounds **1–12** against *E. lanigerum* at 200 μg/mL are shown in Table 5. The CMRs at 72 h of compounds **2**–**12** ranged from 22.7% to 38.1%. In particular, the CMR at 72 h of compound **4** (R as furan-2-yl) was 38.1%, which was 1.6-fold that of compound **1** (72 h CMR: 24.5%). Whereas CMRs at 72 h of compounds **5** (R as pyridin-3-yl) and **6** (R as pyridin-4-yl) were 33.4% and 24.8%, respectively. The CMRs at 72 h of compounds **8–12** (R as benzyl and naphthalen-1-ylmethylene) were 22.7%–32.9%. Obviously, it suggested that introduction of the furan-2-ylcarbonyloxymethylene group at the C-2 position of compound **1** was very important for aphicidal activity against *E. lanigerum*. Based on our previous results [48], it demonstrated that introduction of the CH_2_ between the (un)substituted phenyl and the carbonyl groups was not necessary for aphicidal activity against *E. lanigerum*.

#### 3.2.4. The Effects on the Features of Mite Cuticles by SEM

**Figure 5:**
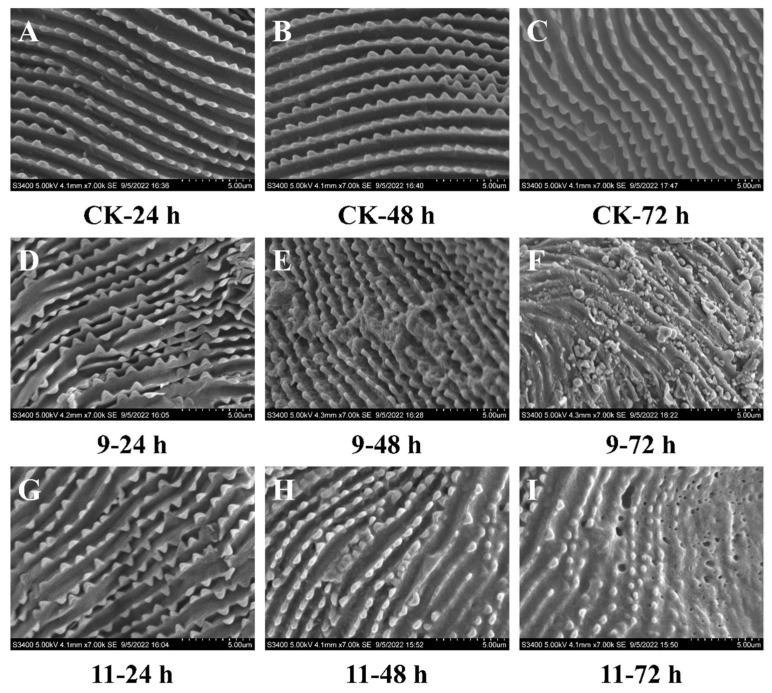
The scanning electron microscope images of *T. cinnabarinus* treated with compounds **9** (**D**–**F**), **11** (**G**–**I**), and aq. Tween-80 (0.1 g/L) (**A**–**C**) after 24, 48, and 72 h, respectively. Bars (**A**–**I**): 5.00 µm.

To investigate the preliminary mechanism of potent compounds **9** and **11** against *T. cinnabarinus*, we conducted an experiment to observe the cuticle symptoms of compound **9**- and **11**-treated *T. cinnabarinus* by using scanning electron microscope (SEM) imaging method (Figure 5). These distinct phenomena were obtained by comparison with the blank control and the treated groups. The arrangement of cuticles in the blank control group (Figure 5A–C) was well regulated and aligned. However, after 24 h, the crests of the mite cuticles of the compound **9**- and **11**-treated groups (Figure 5D,G) began to fracture and appeared irregular. After 48 h (Figure 5E,H), the sectional cuticles of the **9**-treated group were assembled into masses; additionally, the crests of **11**-treated mites partially disappeared. After 72 h, the crests of mite cuticles of the **9**- and **11**-treated groups (Figure 5F,I) all appeared broken and rough and their surfaces were accompanied by the characteristics of collapsed and lacunal symptoms [48,49].

## 4. Conclusions

In this study, by using piperine as a lead compound, we synthesized a series of new ester derivatives of piperine by applying the Vilsmeier–Haack–Arnold reaction under mild conditions. The key steric configurations of compounds **6** and **7** were confirmed by single-crystal X–ray diffraction. Their agricultural bioactivities were evaluated against three crop-threatening pests, *T. cinnabarinus*, *A. citricola*, and *E. lanigerum*. Compounds **9** and **11** showed >45-fold higher acaricidal activity than piperine and exhibited almost 2.6-fold the control efficiency of piperine against *T. cinnabarinus* in the greenhouse. In addition, compound **6** (R as pyridin-4-yl: had >10-fold higher aphicidal activity than that of piperine) displayed the most potent aphicidal activity against *A. citricola*. Compound **4** (R as furan-2-yl) showed 1.6-fold the aphicidal activity of piperine against *E. lanigerum*. The effects of the compounds on the cuticles of *T. cinnabarinus* were observed by the SEM imaging method. These results will pave the way for future applications of piperine and its derivatives as botanical pesticides.

## Figures and Tables

**Table 1:** Acaricidal activity of compounds **1–12** against female adults of *T. cinnabarinus* at 0.5 mg/mL.

Compound	Corrected Mortality Rate (Mean ± SE, %) ^a^	
	48 h	72 h
**1**	8.32 ± 2.3	25.2 ± 3.1 i ^b^
**2**	16.1 ± 3.9	30.9 ± 0.6 hi
**3**	13.7 ± 2.2	33.0 ± 3.2 gh
**4**	18.2 ± 2.8	41.7 ± 1.2 ef
**5**	13.5 ± 2.7	30.4 ± 3.8 hi
**6**	15.3 ± 3.2	45.0 ± 0.8 def
**7**	22.1 ± 3.1	46.3 ± 0.2 de
**8**	18.7 ± 2.8	39.1 ± 0.7 fg
**9**	39.1 ± 3.2	59.4 ± 0.9 b
**10**	32.0 ± 3.6	50.8 ± 2.4 cd
**11**	25.0 ± 1.1	56.3 ± 2.6 bc
**12**	27.4 ± 2.0	41.7 ± 2.5 ef
spirodiclofen	50.4 ± 1.8	88.6 ± 0.9 a

^a^ Average of three replicates. ^b^ Multiple range tests using Duncan’s test (*p* < 0.05). The same letters denote treatments that are not significantly different from each other.

**Table 2:** Toxicity regression analysis of compounds **1**, **9**, and **11** against *T. cinnabarinus* at 72 h.

Compound	LC_50_ (mg/mL)	Confidence Interval 95% (mg/mL)	Regression Equation ^a^	*r*
**1**	14.198	9.545–25.947	Y = –0.735 + 0.638X	0.990
**9**	0.298	0.243–0.368	Y = 0.656 + 1.247X	0.996
**11**	0.313	0.252–0.395	Y = 0.614 + 1.216X	0.997
spirodiclofen	0.115	0.093–0.141	Y = 1.147 + 1.220X	0.990

^a^ Regression analysis by IBM SPSS Statistics 20.0, *p* < 0.05.

**Table 3:** Aphicidal activity of compounds **1–12** against apterous adults of *A. citricola* at 0.04 μg/nymph.

Compound	Corrected Mortality Rate (Mean ± SE, %) ^a^	
	24 h	48 h
**1**	7.8 ± 1.1	25.0 ± 1.9 e ^b^
**2**	6.7 ± 1.9	18.2 ± 1.9 f
**3**	8.9 ± 1.1	27.3 ± 3.0 e
**4**	16.7 ± 1.9	29.5 ± 2.2 de
**5**	15.6 ± 1.1	28.4 ± 1.9 de
**6**	35.6 ± 1.1	55.7 ± 1.9 b
**7**	15.6 ± 2.2	35.2 ± 3.4 cd
**8**	20.0 ± 1.9	29.5 ± 3.0 de
**9**	11.1 ± 1.1	35.2 ± 1.9 cd
**10**	16.7 ± 1.9	37.5 ± 3.0 c
**11**	14.4 ± 1.1	39.8 ± 1.1 c
**12**	30.0 ± 1.9	42.0 ± 1.9 c
methomyl ^c^	50.0 ± 1.9	88.6 ± 1.1 a

^a^ Average of three replicates. ^b^ Multiple range tests using Duncan’s test (*p* < 0.05). The same letters denote treatments that are not significantly different from each other. ^c^ 0.004 μg/nymph.

**Table 4:** LD_50_ values of compounds **1** and **6** against *A. citricola* at 48 h.

Compound	LD_50_ (µg/nymph)	Confidence interval 95% (µg/nymph)	Regression Equation ^a^	*r*
**1**	0.308	0.217–0.491	Y = 0.419 + 0.821X	0.997
**6**	0.030	0.024–0.037	Y = 2.366 + 1.549X	0.982

^a^ Regression analysis by IBM SPSS Statistics 20.0, *p* < 0.05.

**Table 5:** Aphicidal activity of compounds **1–12** against *E. lanigerum* at 200 μg/mL.

Compound	Corrected Mortality Rate (Mean ± SE, %) ^a^	
	48 h	72 h
**1**	16.7 ± 1.4	24.5 ± 1.0 e ^b^
**2**	11.0 ± 1.8	22.9 ± 2.3 e
**3**	10.7 ± 2.0	29.9 ± 0.5 cd
**4**	23.9 ± 2.2	38.1 ± 0.6 b
**5**	11.1 ± 3.3	33.4 ± 2.1 c
**6**	12.3 ± 1.8	24.8 ± 1.8 e
**7**	13.2 ± 2.1	22.8 ± 0.7 e
**8**	15.2 ± 1.9	32.4 ± 2.6 c
**9**	17.7 ± 1.4	32.9 ± 0.5 c
**10**	17.3 ± 2.5	25.9 ± 2.2 de
**11**	13.7 ± 2.8	29.7 ± 1.6 cd
**12**	12.7 ± 1.1	22.7 ± 0.6 e
thiamethoxam	50.2 ± 0.2	90.9 ± 1.3 a
chlorpyrifos	48.8 ± 0.8	91.3 ± 0.6 a

^a^ Average of three replicates. ^b^ Multiple range tests using Duncan’s test (*p* < 0.05). The same letters denote treatments that are not significantly different from each other.

## Data Availability

The dataset utilized in this study is available upon request.

## Supplementary Materials

The following supporting information can be downloaded at: https://www.mdpi.com/article/10.3390/insects14010040/s1, Figures S1–S5: The photographs of compounds **1**, **9**, **11**, spirodiclofen, and the blank control groups for control effect evaluation. Structural characterization data (IR, ^1^HNMR, ^13^CNMR and melting points) of all compounds. ^1^H NMR and ^13^C NMR spectra of all compounds (PDF).
