# Supplementary material for: Synthesis of Piperine-Based Ester Derivatives with Diverse Aromatic Rings and Their Agricultural Bioactivities against Tetranychus cinnabarinus Boisduval, Aphis citricola Van der Goot, and Eriosoma lanigerum Hausmann
Source: Insects. 2022 Dec 31;14(1):40. doi: 10.3390/insects14010040 (PMC9862344; doi:10.3390/insects14010040)
Supplement: Supplementary file 1 [file insects-14-00040-s001.zip › insects-2027782-supplementary.pdf]

Supporting Information

Synthesis of Piperine-Based Ester Derivatives with Diverse Aromatic Rings and Their Agricultural Bioactivities against *Tetranychus cinnabarinus* Boisduval, *Aphis citricola* Van der Goot, and *Eriosoma lanigerum* Hausmann

Tianze Li[1], Min Lv[1,*], Houpeng Wen[1], Yanyan Wang[1], Sunita Thapa[1], Shaoyong Zhang[2], and Hui Xu[1,*]

[1] College of Plant Protection, Northwest A&F University, Yangling 712100, China

[2] Key Laboratory of Vector Biology and Pathogen Control of Zhejiang Province, College of Life Science, Huzhou University, Huzhou 313000, China

* Correspondence: lvmin@nwsuaf.edu.cn (M.L.); orgxuhui@nwsuaf.edu.cn (H.X.)

Contents

1. Characterization……………………………………………………….2

2. ^{1}H NMR spectra of compounds………………………………………3-8

3. ^{13}C NMR spectra of all compounds.

4. Pictures of control effects against *T. cinnabarinus*……………………….9-13

1. Characterization

Data for 6: Yield: 57%; white solid; mp 96-98 ºC; IR cm^{-1} (KBr): 3430, 3303, 3083, 2371, 1723, 1573, 1492, 1369, 951, 648, 539; ^{1}H NMR (400 MHz, $CDCl_3$) δ: 8.79 (d, J = 3.6 Hz, 1H), 7.83-7.85 (m, 2H), 6.90 (s, 1H), 6.85 (d, J = 8.0 Hz, 1H), 6.76-6.78 (m, 1H), 6.52-6.66 (m, 2H), 6.49 (d, J = 10.4 Hz, 1H), 5.98 (d, J = 2.0 Hz, 2H), 5.11 (s, 2H), 3.71 (s, 2H), 3.44 (t, J = 4.8 Hz, 2H), 1.63 (s, 4H), 1.44 (s, 2H). ^{13}C NMR (100 MHz, $CDCl_3$) δ: 167.18, 164.76, 150.65, 148.25, 148.18, 137.20, 137.18, 132.35, 130.77, 129.99, 122.86, 122.35, 121.67, 108.55, 105.59, 101.33, 67.56, 47.76, 42.53, 26.67, 25.82, 24.42.

Data for 7: Yield: 50%; orange solid; mp 179-180 ºC; IR cm^{-1} (KBr): 3304, 3084, 2940, 2860, 2375, 1702, 1594, 1492, 1449, 1264, 1134, 1027, 957, 817, 769, 613, 492; ^{1}H NMR (400 MHz, $CDCl_3$) δ: 6.90 (s, 1H), 6.84 (d, J = 8.0 Hz, 1H), 6.77 (d, J = 7.6 Hz, 1H), 6.54-6.63 (m, 2H), 6.44 (d, J = 6.0 Hz, 1H), 5.97 (s, 2H), 4.97 (s, 2H), 4.80 (s, 2H), 4.42 (s, 2H), 4.22 (s, 5H), 3.76 (s, 2H), 3.51 (s, 2H), 1.67 (s, 4H), 1.53 (s, 2H). ^{13}C NMR (100 MHz, $CDCl_3$) δ: 171.39, 148.19, 147.96, 136.15, 131.02, 130.65, 122.15, 122.10, 108.50, 105.58, 101.26, 71.45, 70.73, 70.16, 69.87, 65.74, 47.82, 42.47, 26.75, 25.92, 24.54.

Data for 8: Yield: 44%; colorless oil; IR cm^{-1} (KBr): 3049, 2937, 2865, 2351, 2186, 1732, 1594, 1491, 1445, 1359, 1250, 1142, 1032, 965, 923, 789, 728, 629, 533; ^{1}H NMR (400 MHz, $CDCl_3$) δ: 8.00 (d, J = 8.4 Hz, 1H), 7.86 (d, J = 8.0 Hz, 1H), 7.77-7.79 (m, 1H), 7.46-7.54 (m, 2H), 7.39-7.42 (m, 2H), 6.83 (d, J = 1.2 Hz, 1H), 6.75-6.78 (m, 1H), 6.74 (d, J = 8.0 Hz, 1H), 6.35-6.46 (m, 2H), 6.15 (d, J = 10.0 Hz, 1H), 5.92 (s, 2H), 4.82 (s, 2H), 4.08 (s, 2H), 3.48 (s, 2H), 2.93 (s, 2H), 1.50 (s, 4H), 1.24 (s, 2H). ^{13}C NMR (100 MHz, $CDCl_3$) δ: 170.98, 167.14, 148.14, 147.94, 136.28, 133.81, 132.09, 130.99, 130.88, 130.66, 130.28, 128.66, 128.16, 128.10, 126.51, 125.86, 125.49, 123.81, 122.08, 121.75, 108.45, 105.48, 101.23, 66.66, 47.28, 42.15, 39.25, 26.39, 25.71, 24.34.

Data for 9: Yield: 56%; yellow oil; IR cm^{-1} (KBr): 3303, 3029, 2936, 2862, 2350, 1734, 1596, 1491, 1446, 1356, 1250, 1142, 1032, 968, 929, 807, 712, 610, 532; ^{1}H NMR (400 MHz, $CDCl_3$) δ: 7.28-7.34 (m, 5H), 6.87 (s, 1H), 6.82 (d, J = 8.0 Hz, 1H), 6.76 (d, J = 7.6 Hz, 1H), 6.45-6.54 (m, 2H), 6.28 (d, J = 8.4 Hz, 1H), 5.95 (s, 2H), 4.83 (s, 2H), 3.64 (s, 4H), 3.22 (s, 2H), 1.59 (s, 4H), 1.40 (s, 2H). ^{13}C NMR (100 MHz, $CDCl_3$) δ: 171.08, 167.30, 148.21, 148.02, 136.47, 133.78, 131.22, 130.94, 130.80, 129.35, 128.65, 127.19, 122.18, 121.88, 108.51, 105.57, 101.29, 66.70, 47.57, 42.36, 41.44, 26.57, 25.81, 24.47.

Data for 10: Yield: 54%; colorless oil; IR cm^{-1} (KBr): 3305, 2935, 2861, 2373, 2240, 1736, 1613, 1499, 1445, 1355, 1248, 1143, 1032, 967, 928, 806, 727, 607, 520; ^{1}H NMR (400 MHz, $CDCl_3$) δ: 7.26 (dd, J_1 = 8.4 Hz, J_2 = 5.6 Hz, 2H), 7.03 (t, J = 8.4 Hz, 2H), 6.88 (s, 1H), 6.82 (d, J = 8.0 Hz, 1H), 6.76 (d, J = 8.0 Hz, 1H), 6.46-6.56 (m, 2H), 6.29 (d, J = 8.8 Hz, 1H), 5.96 (d, J = 2.0 Hz, 2H), 4.83 (s, 2H), 3.65 (s, 2H), 3.61 (s, 2H), 3.28 (t, J = 4.8 Hz, 2H), 1.61 (s, 4H), 1.43 (s, 2H). ^{13}C NMR (100 MHz, $CDCl_3$) δ: 170.92, 167.23, 163.28, 160.84, 148.20, 148.05, 136.60, 131.37, 130.96, 130.88, 130.86, 130.62, 129.50, 129.47, 122.22, 121.78, 115.57, 115.36, 108.50, 105.55, 101.28,

66.76, 47.61, 42.39, 40.47, 26.59, 25.78, 24.47.

Data for 11: Yield: 53%; colorless oil; IR cm^{-1} (KBr): 3303, 2936, 2865, 2420, 1735, 1588, 1491, 1446, 1357, 1249, 1146, 1031, 967, 930, 729, 522; ^{1}H NMR (400 MHz, $CDCl_3$) δ: 7.31 (d, J = 8.4 Hz, 2H), 7.22 (d, J = 8.4 Hz, 2H), 6.88 (d, J = 1.2 Hz, 1H), 6.81-6.83 (m, 1H), 6.77 (d, J = 8.0 Hz, 1H), 6.45-6.55 (m, 2H), 6.27 (d, J = 8.8 Hz, 1H), 5.96 (s, 2H), 4.83 (s, 2H), 3.64 (s, 2H), 3.61 (s, 2H), 3.22-3.23 (m, 1H), 1.61 (s, 4H), 1.42 (t, J = 4.0 Hz, 2H). ^{13}C NMR (100 MHz, $CDCl_3$) δ: 170.62, 167.20, 148.20, 148.05, 136.62, 133.19, 132.22, 131.37, 130.86, 130.75, 130.55, 128.76, 122.23, 121.77, 108.51, 105.56, 101.28, 66.82, 47.58, 42.39, 40.69, 26.58, 25.78, 24.46.

Data for 12: Yield: 44%; colorless oil; IR cm^{-1} (KBr): 3309, 2936, 2857, 2239, 1735, 1618, 1501, 1446, 1355, 1247, 1140, 1031, 968, 926, 808, 727, 606, 526; ^{1}H NMR (400 MHz, $CDCl_3$) δ: 7.20 (d, J = 8.8 Hz, 2H), 6.87 (s, 1H), 6.86 (d, J = 8.4 Hz, 2H), 6.79-6.82 (m, 1H), 6.76 (d, J = 8.0 Hz, 1H), 6.45-6.54 (m, 2H), 6.26 (dd, J_1 = 6.8 Hz, J_2 = 3.2 Hz, 1H), 5.95 (s, 2H), 4.82 (s, 2H), 3.78 (s, 3H), 3.64 (s, 2H), 3.58 (s, 2H), 3.24 (s, 2H), 1.60 (s, 4H), 1.42 (s, 2H). ^{13}C NMR (100 MHz, $CDCl_3$) δ: 171.38, 167.28, 158.77, 148.18, 147.99, 136.41, 131.07, 130.92, 130.84, 130.36, 125.82, 122.15, 121.85, 114.02, 108.49, 105.54, 101.26, 66.53, 55.25, 47.57, 42.34, 40.50, 26.57, 25.79, 24.47.

2. ^{1}H NMR spectra of compounds

compound **2**

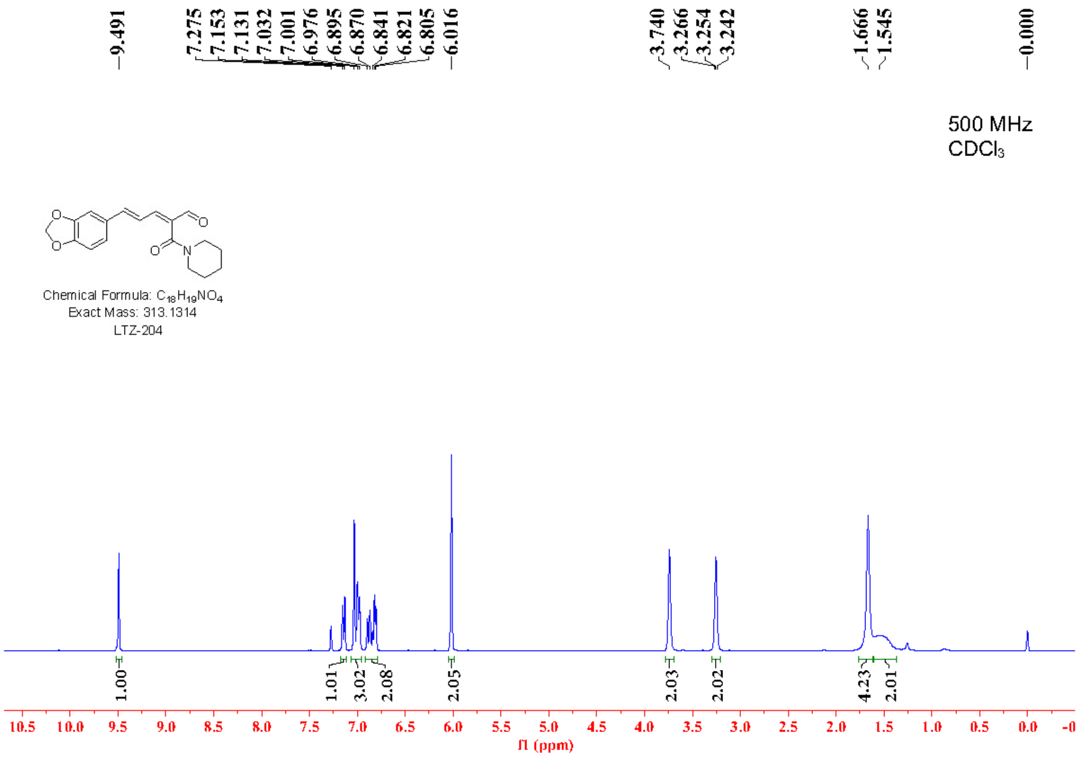

compound **3**

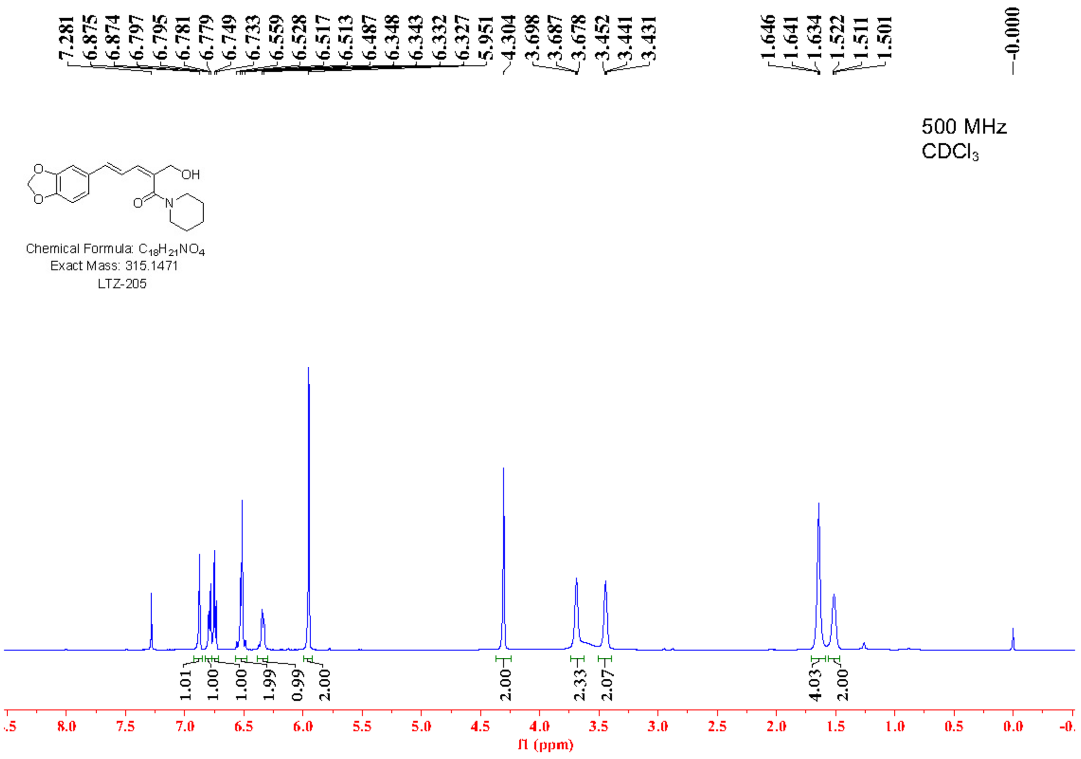

compound **4**

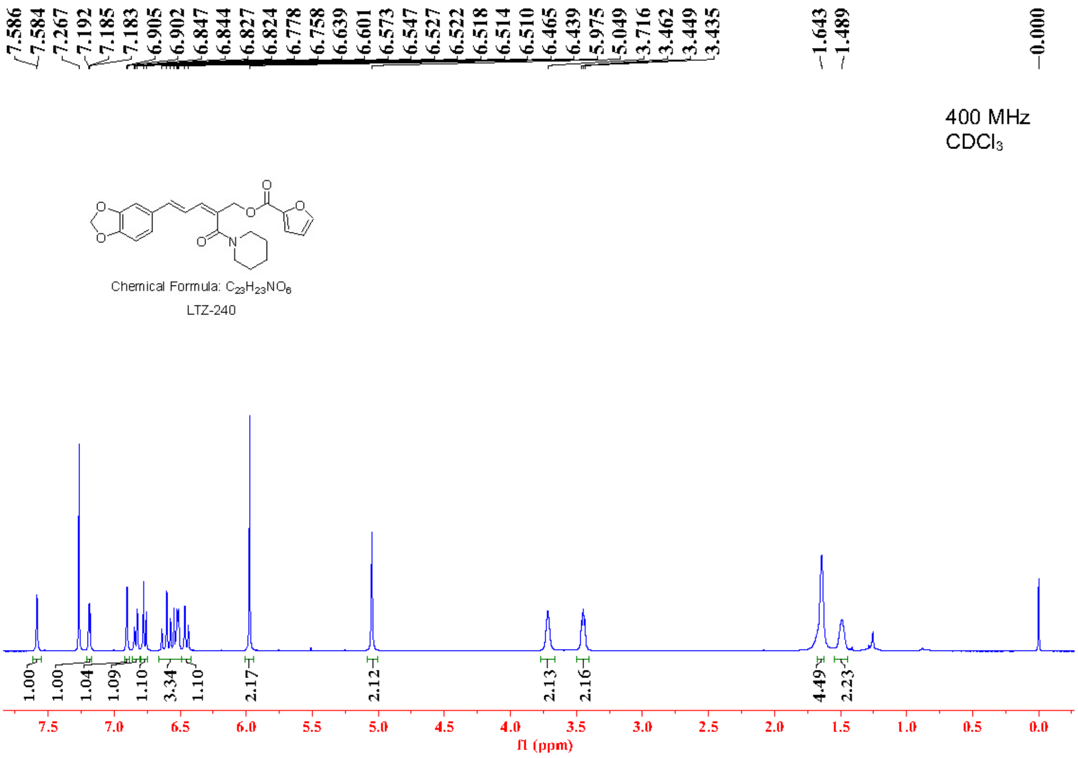

compound **5**

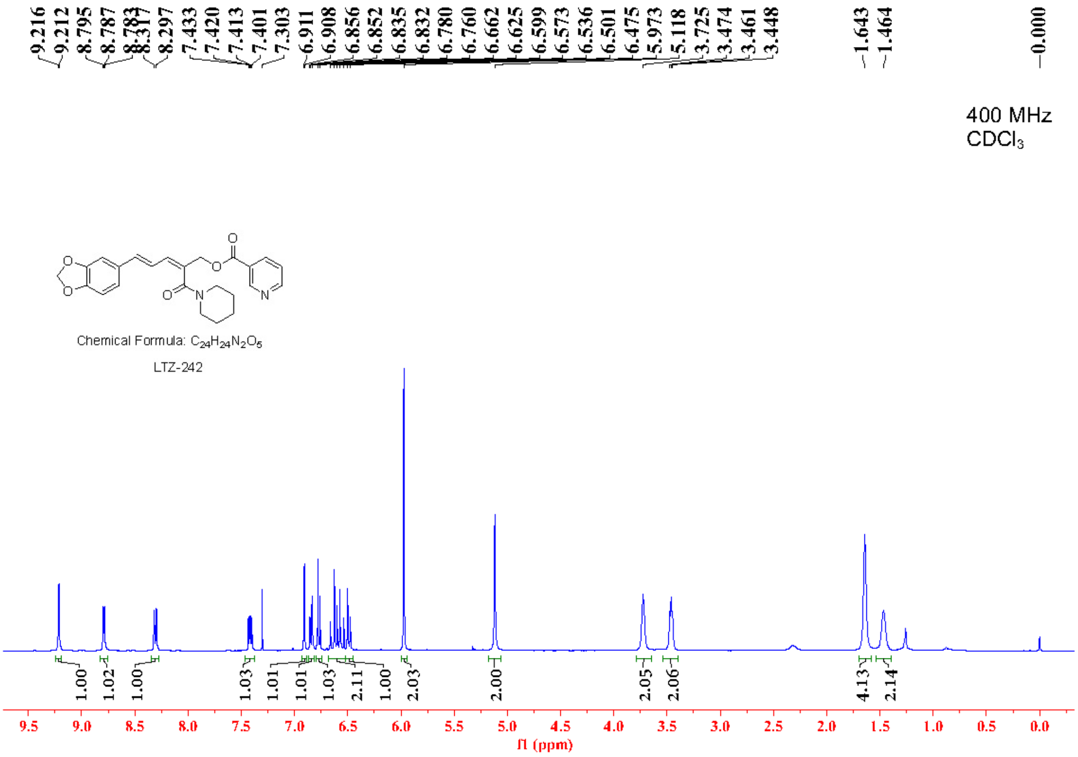

compound **6**

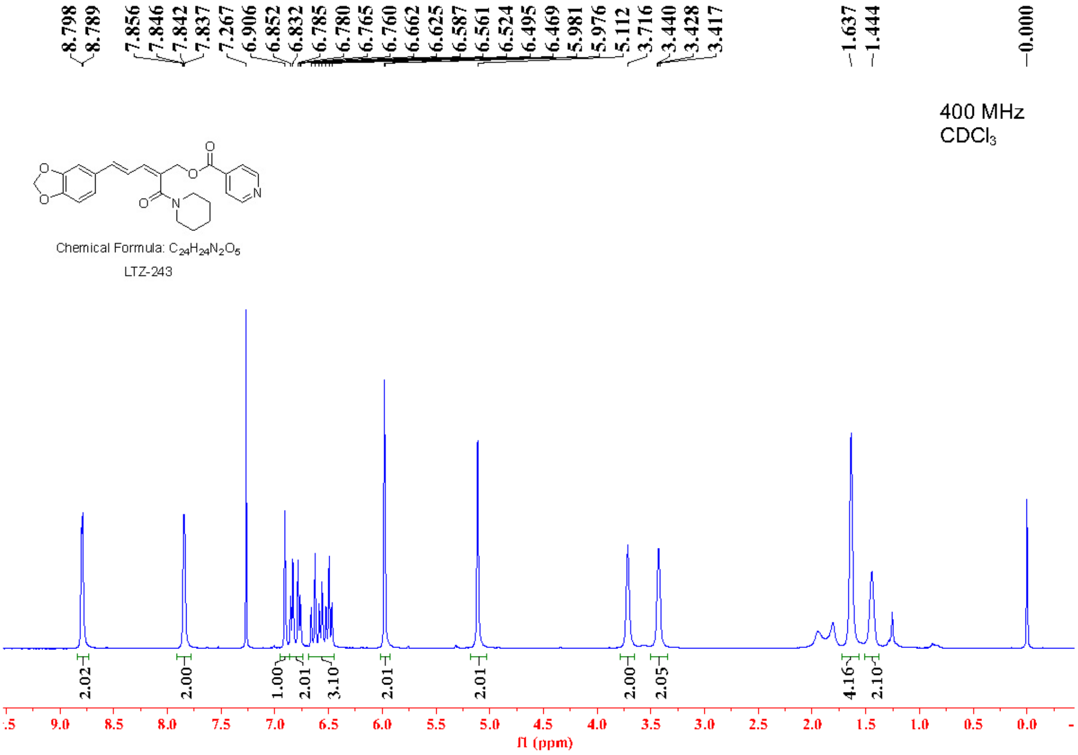

compound **7**

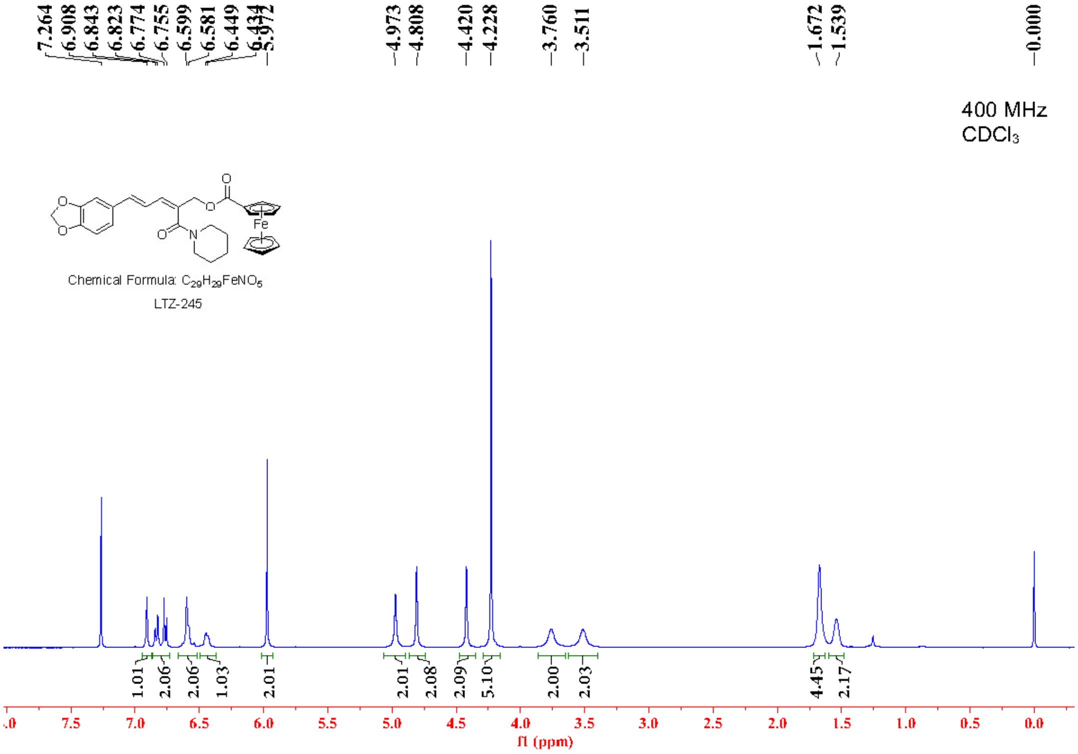

compound **8**

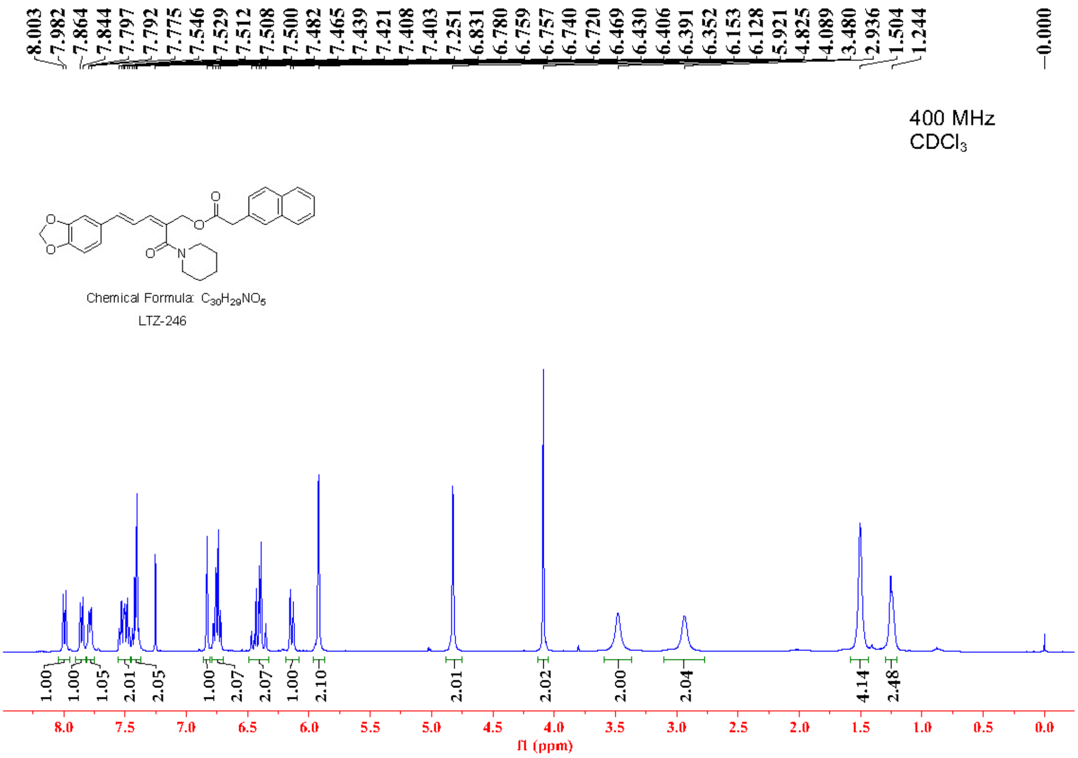

compound **9**

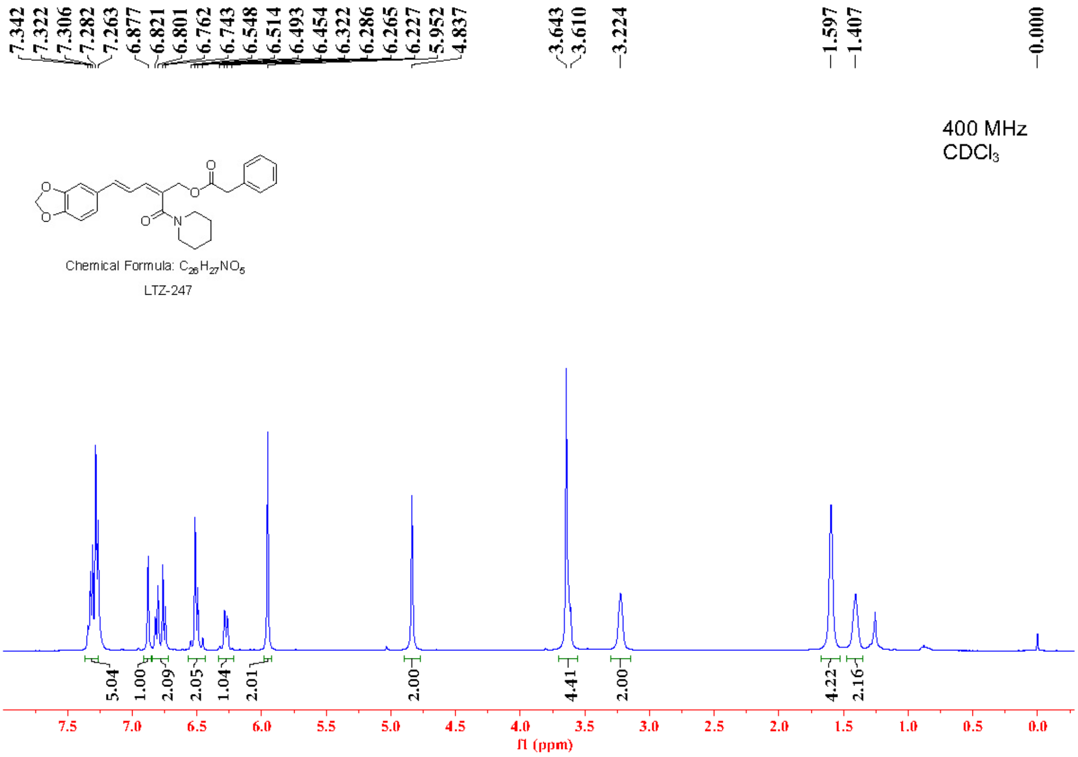

compound **10**

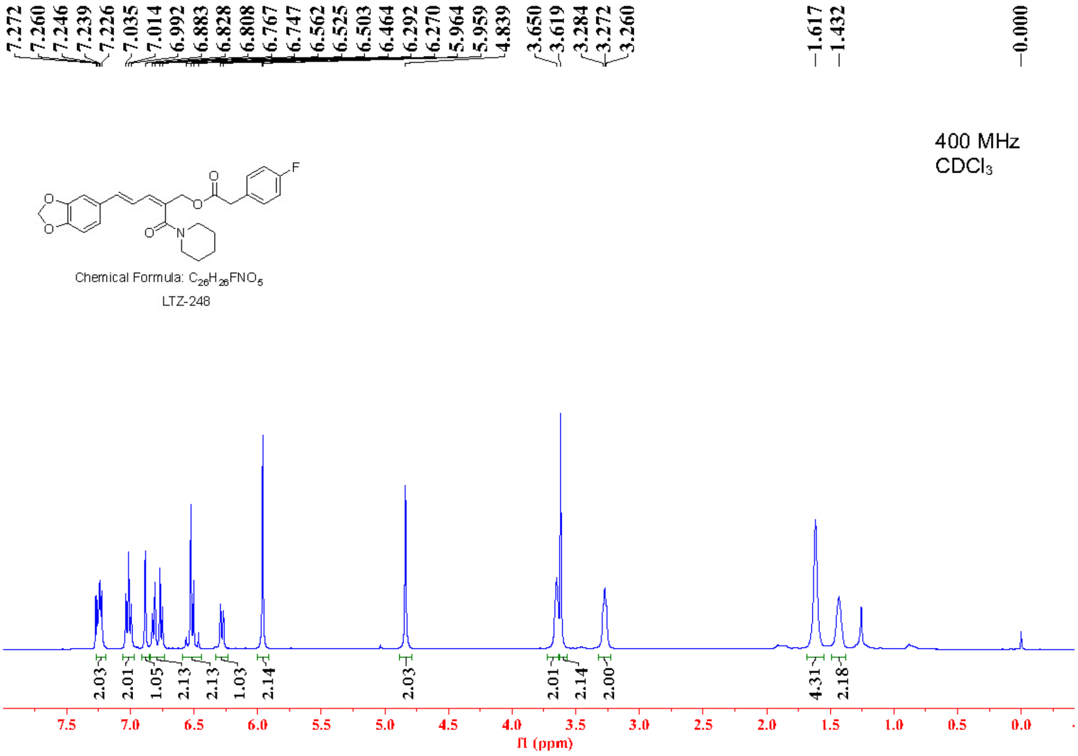

compound **11**

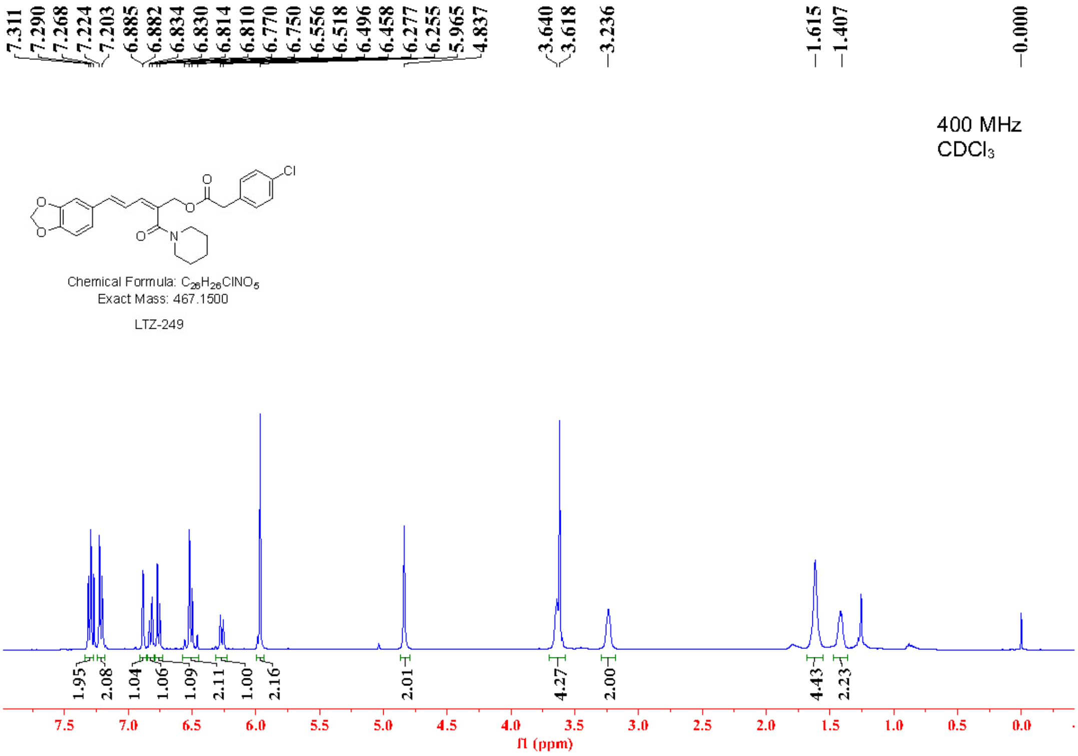

compound **12**

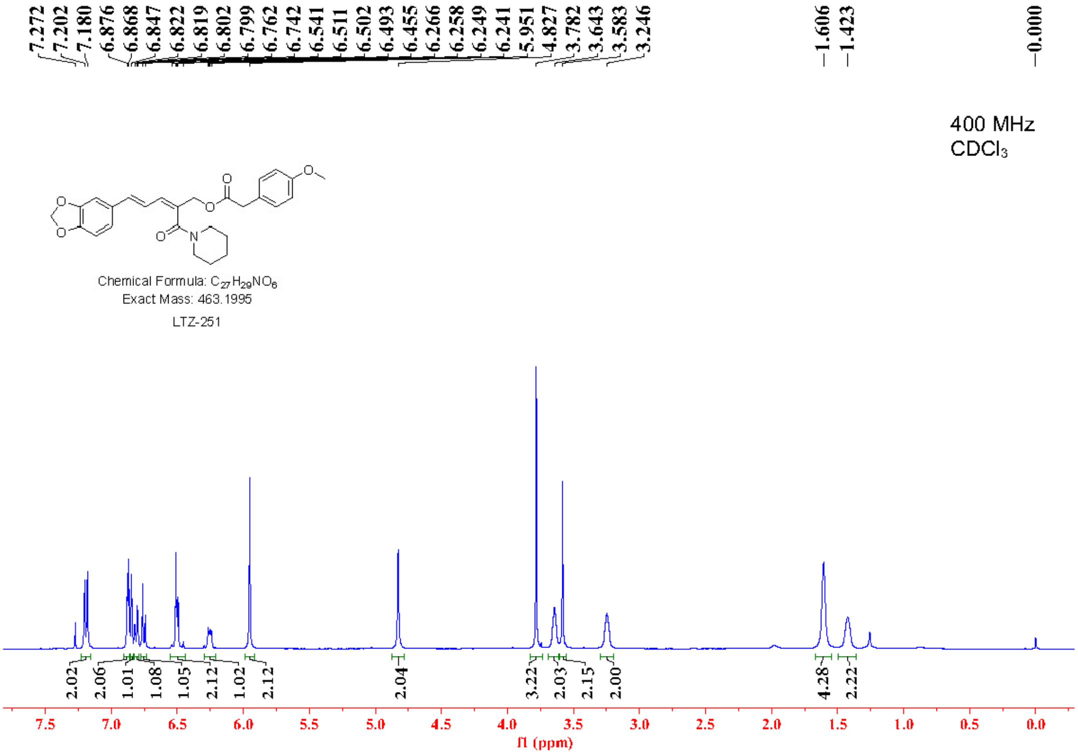

400 MHz
CDCl3
Chemical Formula: C27H29NO6
Exact Mass: 463.1995
LTZ-251
7.272
7.202
7.180
6.876
6.868
6.847
6.822
6.819
6.802
6.799
6.762
6.742
6.541
6.511
6.502
6.493
6.455
6.266
6.258
6.249
6.241
5.951
4.827
3.782
3.643
3.583
3.246
1.606
1.423
0.000
2.02
2.06
1.01
1.08
1.05
2.12
1.02
2.12
2.04
3.22
2.03
2.15
2.00
4.28
2.22
fl (ppm)

3. ^{13}C NMR spectra of all compounds.

^{13}C NMR spectrum of compound **2**

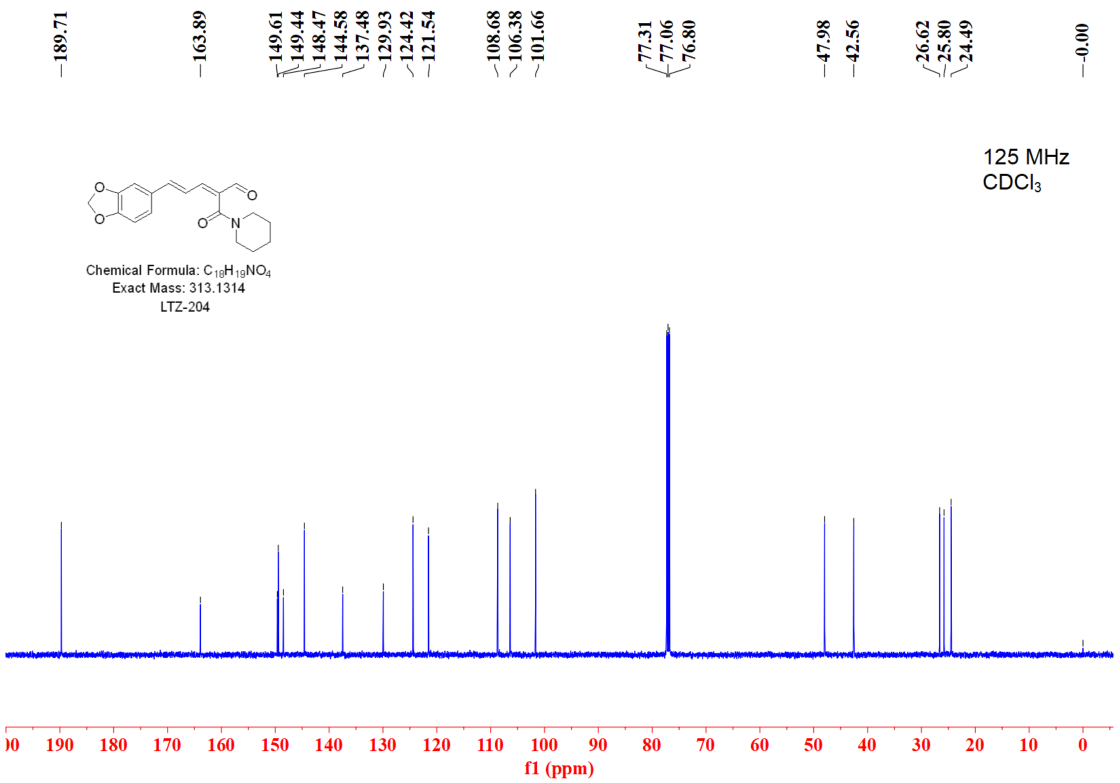

^{13}C NMR spectrum of compound **3**

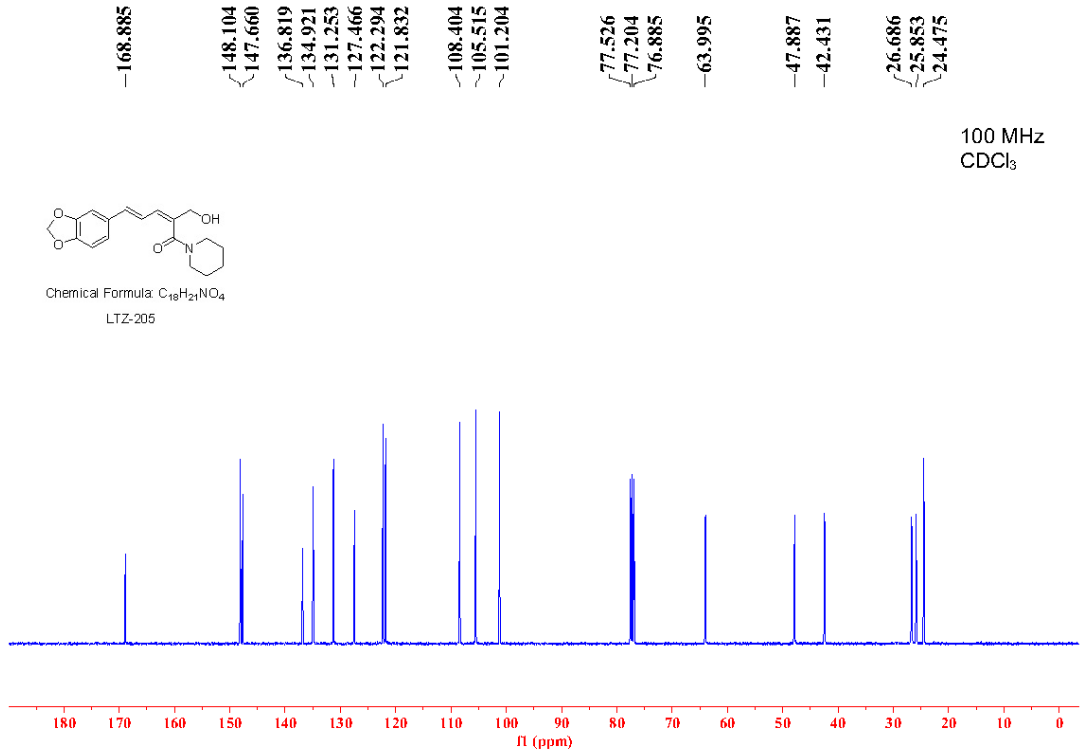

^{13}C NMR spectrum of compound **4**

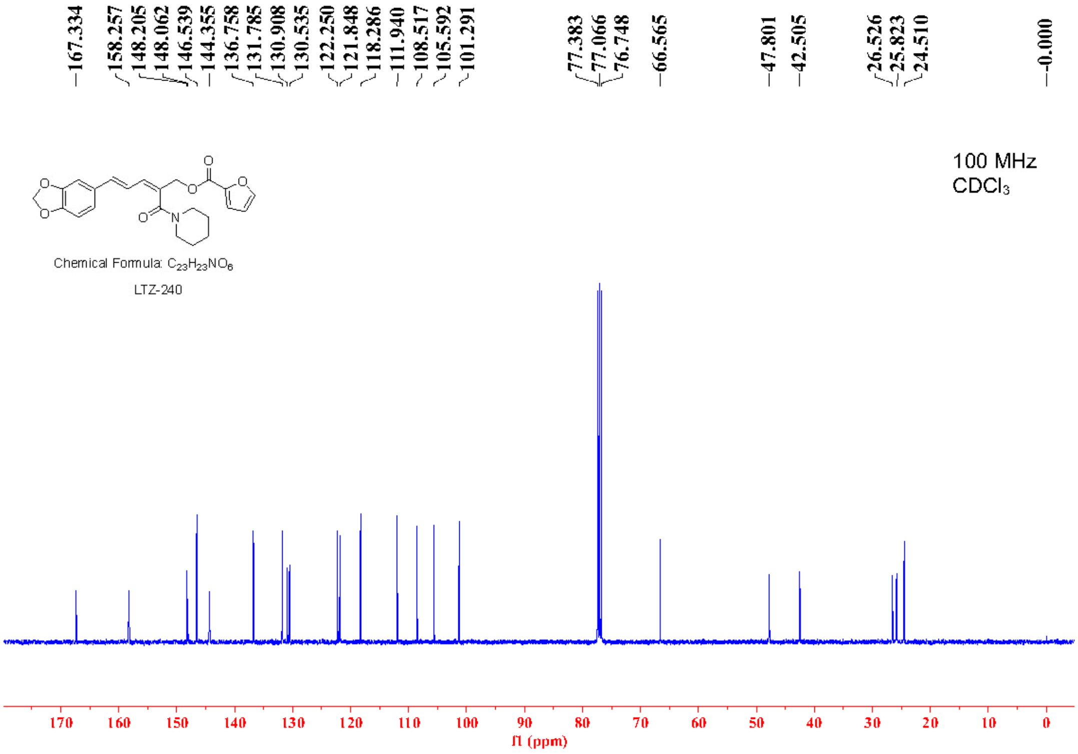

^{13}C NMR spectrum of compound **5**

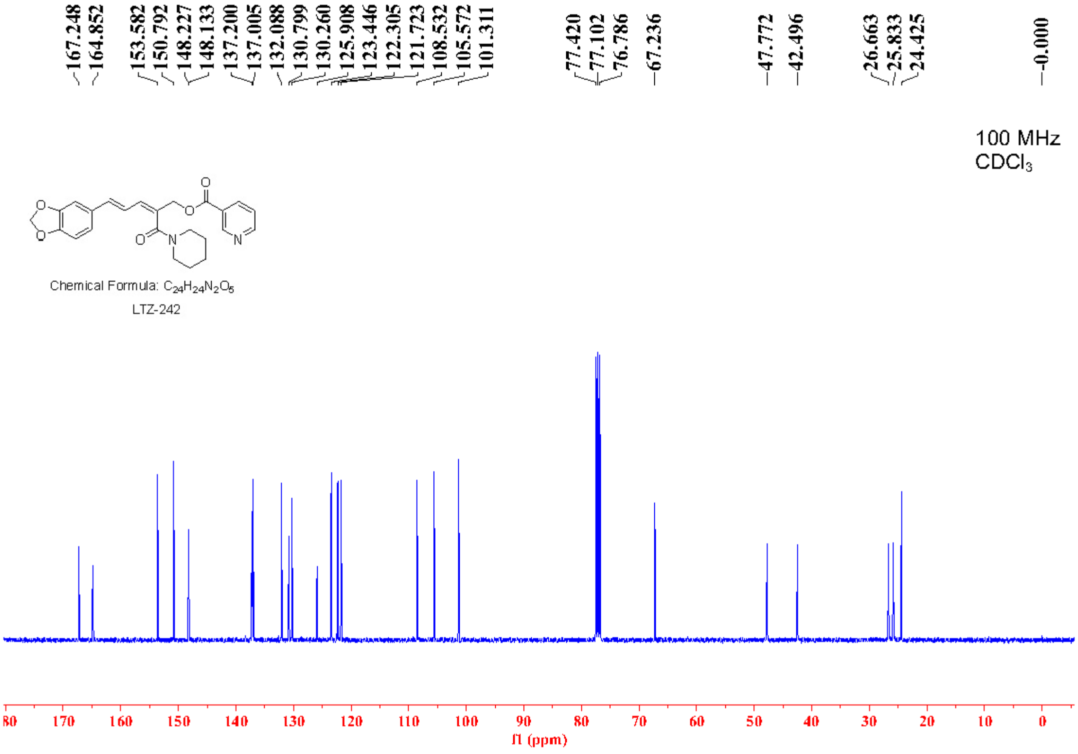

^{13}C NMR spectrum of compound **6**

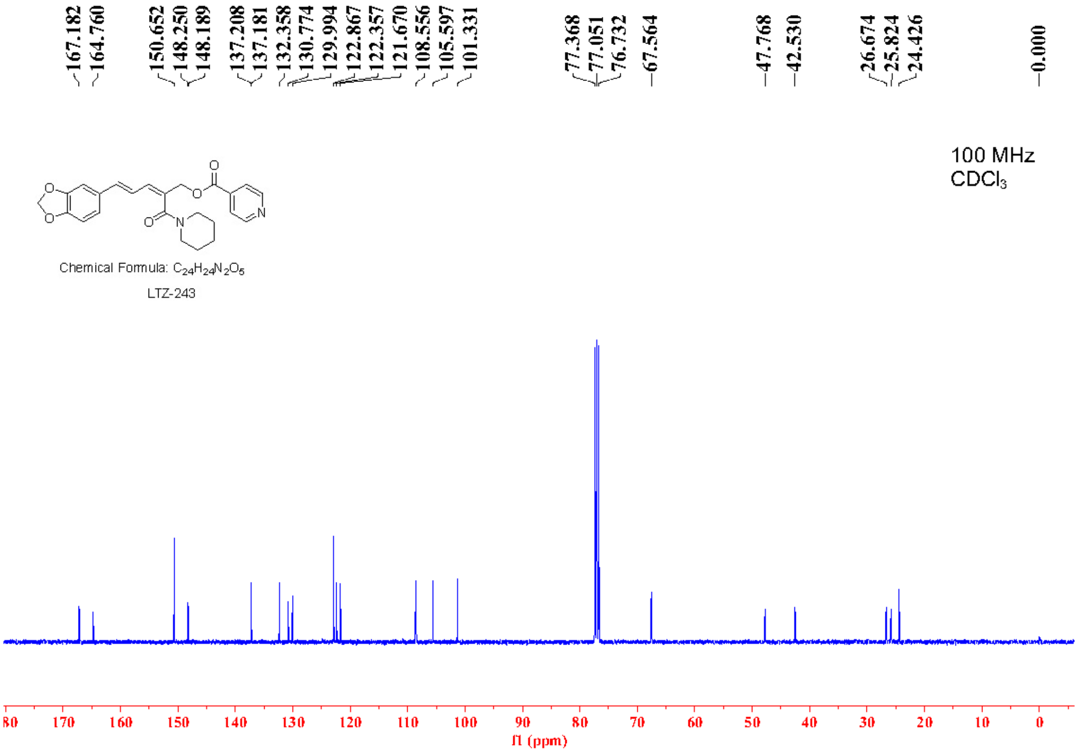

^{13}C NMR spectrum of compound **7**

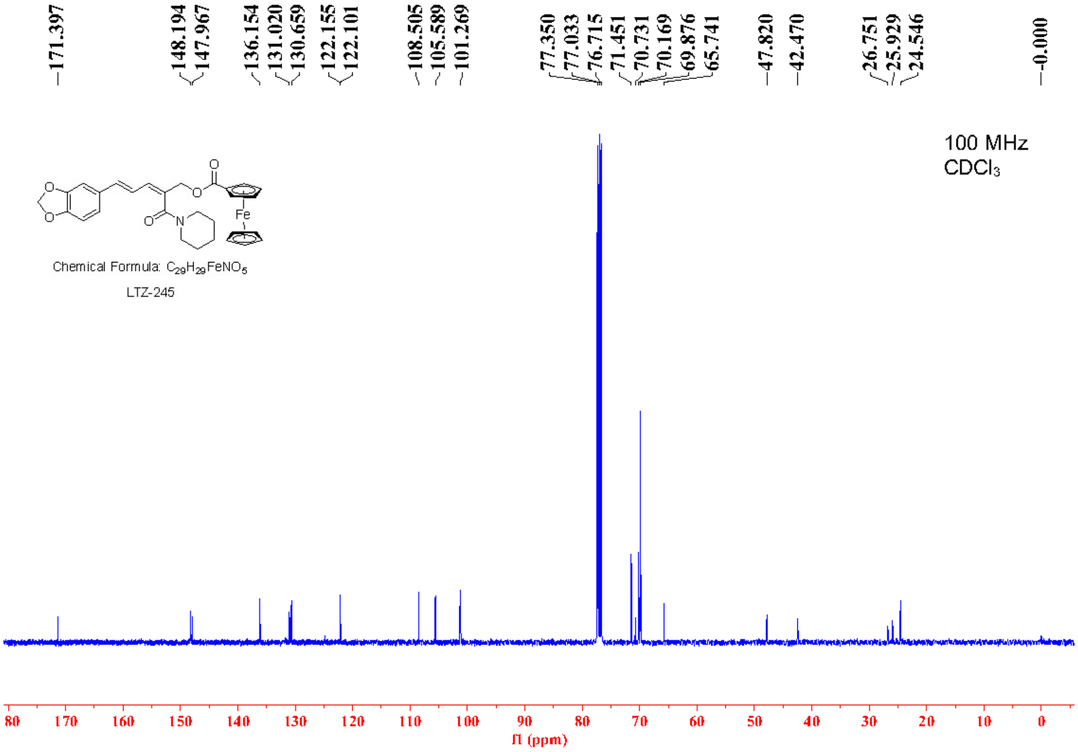

^{13}C NMR spectrum of compound **8**

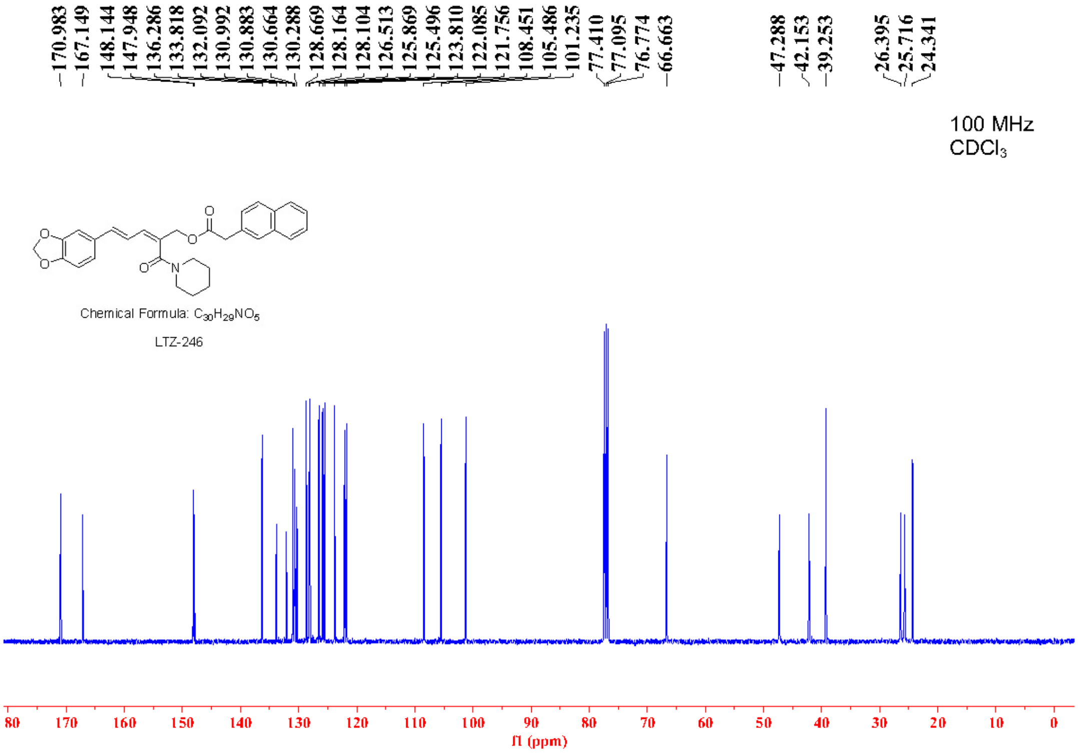

^{13}C NMR spectrum of compound **9**

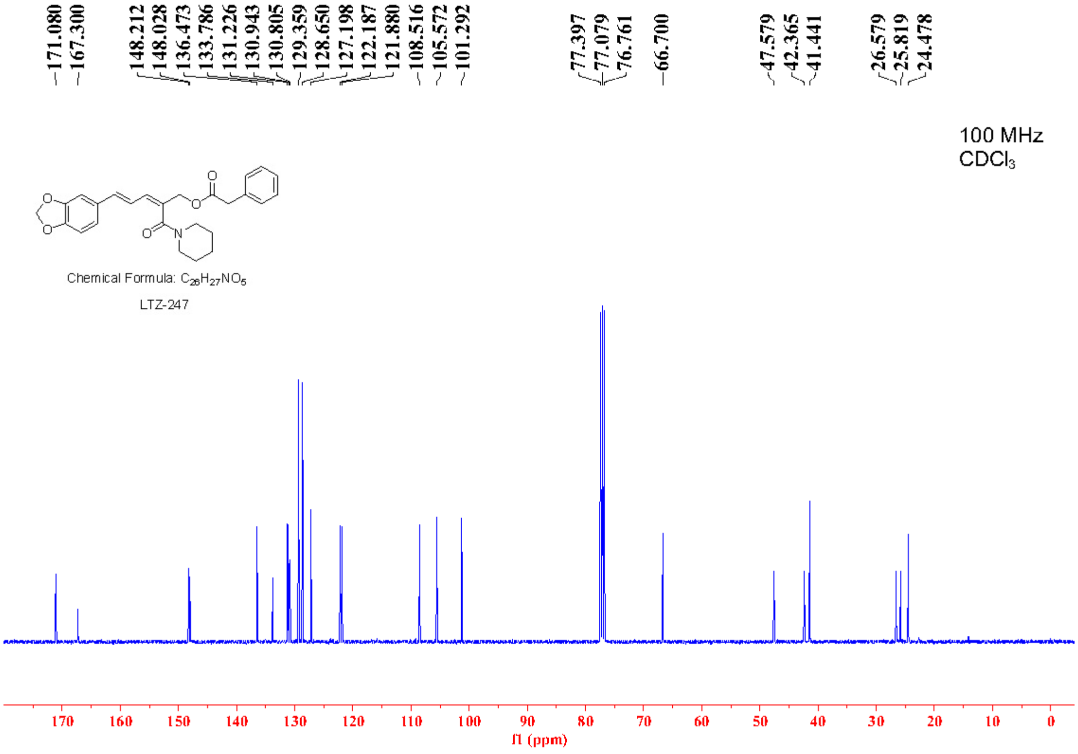

^{13}C NMR spectrum of compound **10**

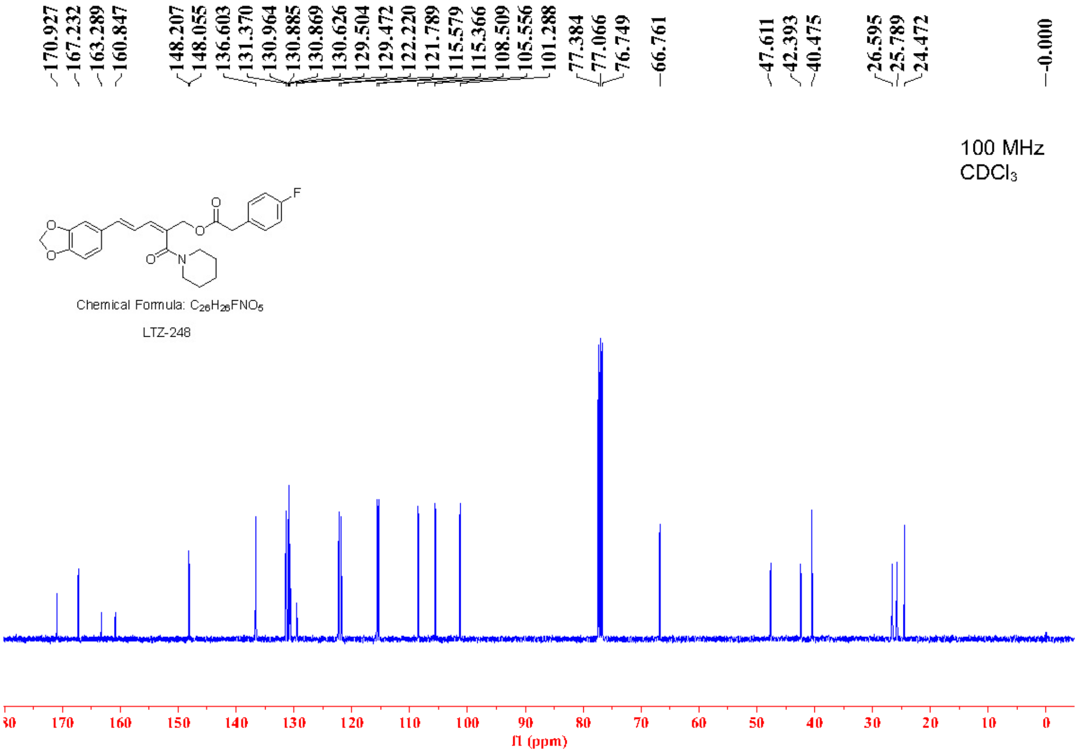

^{13}C NMR spectrum of compound **11**

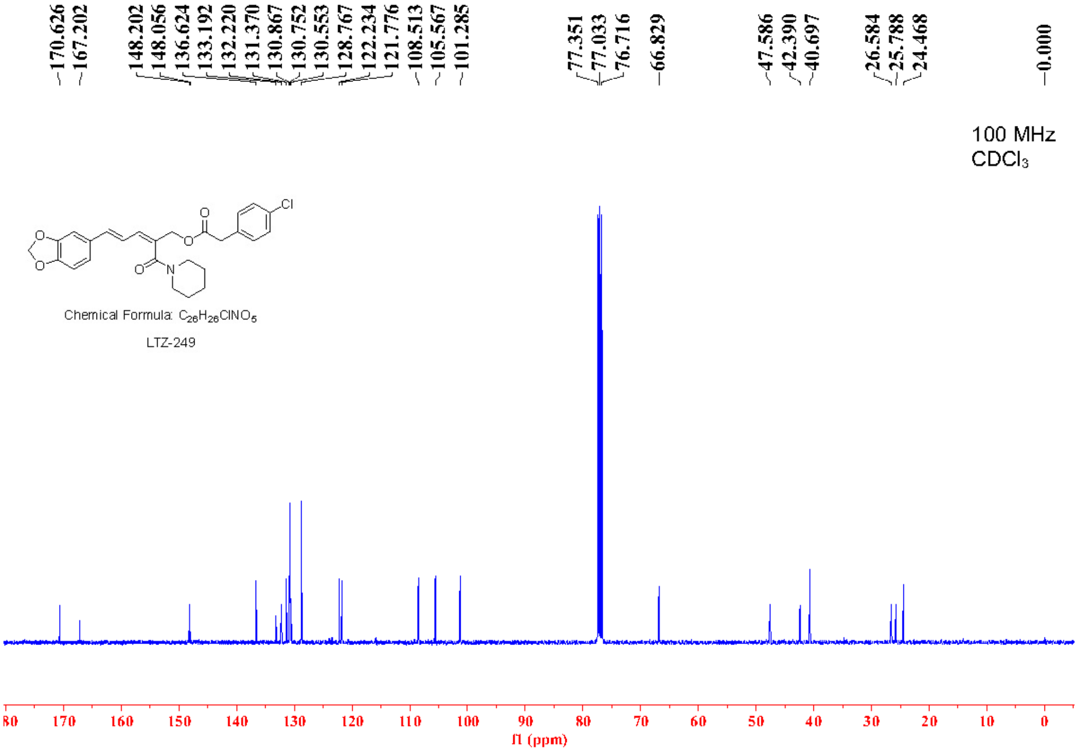

^{13}C NMR spectrum of compound 12

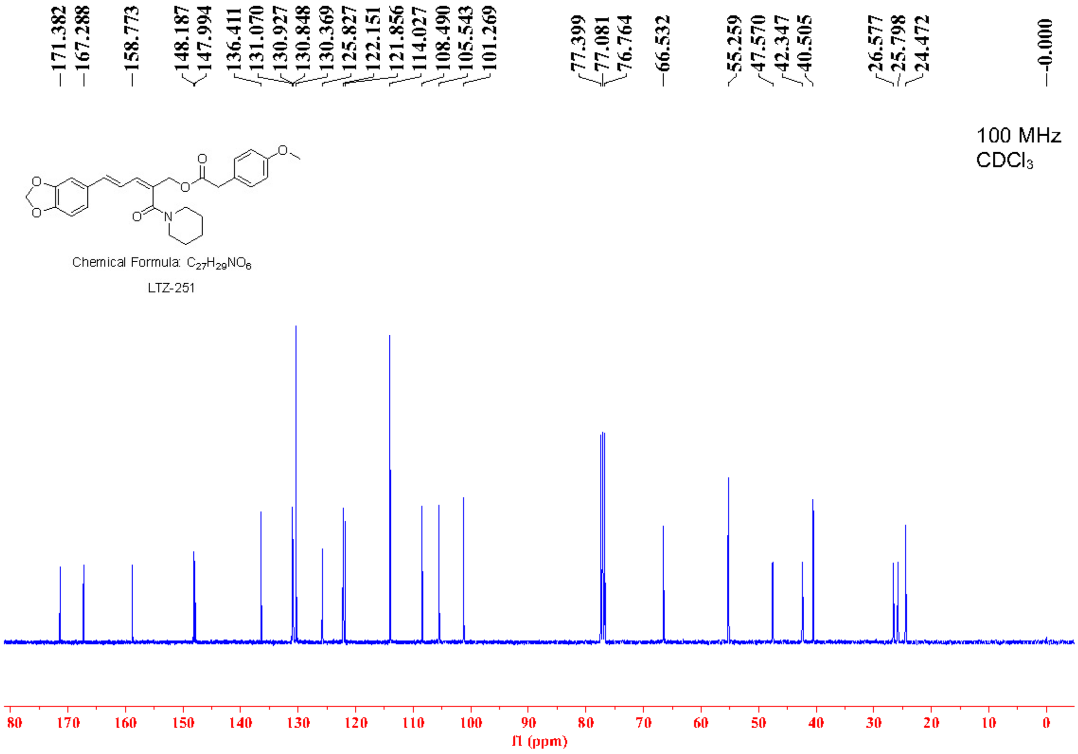

4. Pictures of control effects against *T. cinnabarinus*

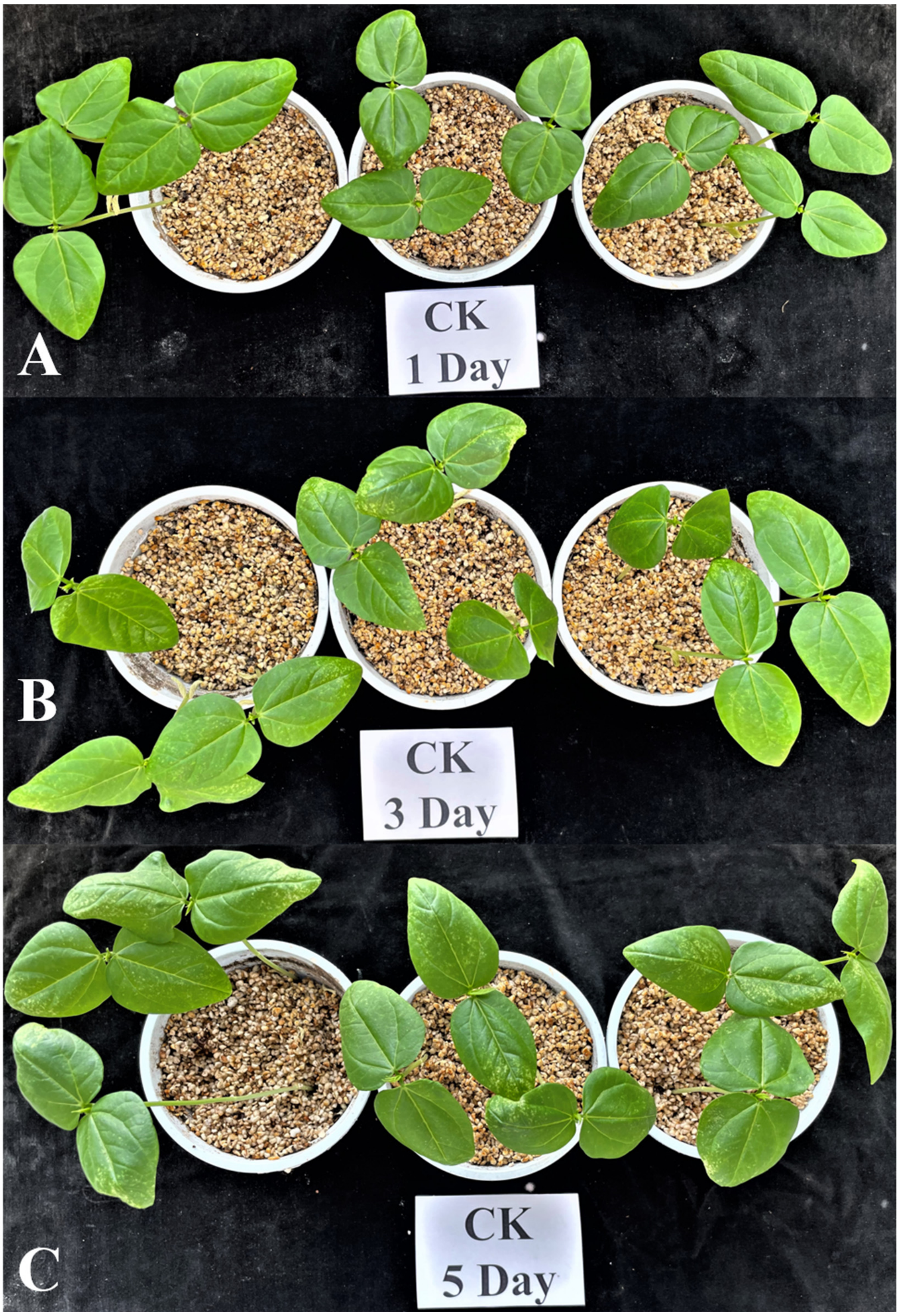

Figure S1. Pictures of control efficiency of the control group after 1st day (A), 3rd day (B), and 5th day (C) against *T. cinnabarinus* in the greenhouse.

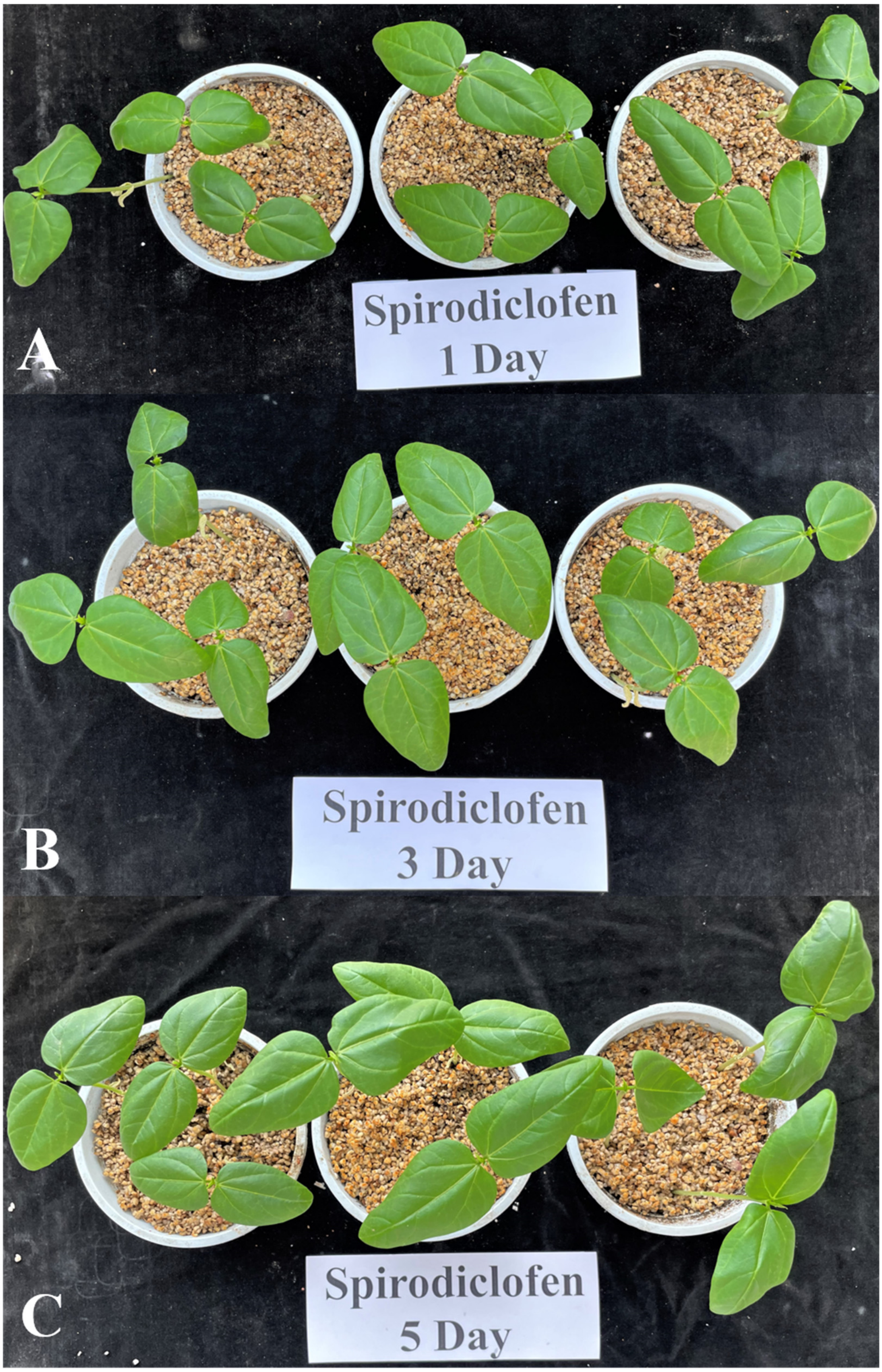

Figure S2. Pictures of control efficiency of positive control group spirodiclofen after 1st day (A), 3rd day (B), and 5th day (C) against *T. cinnabarinus* in the greenhouse.

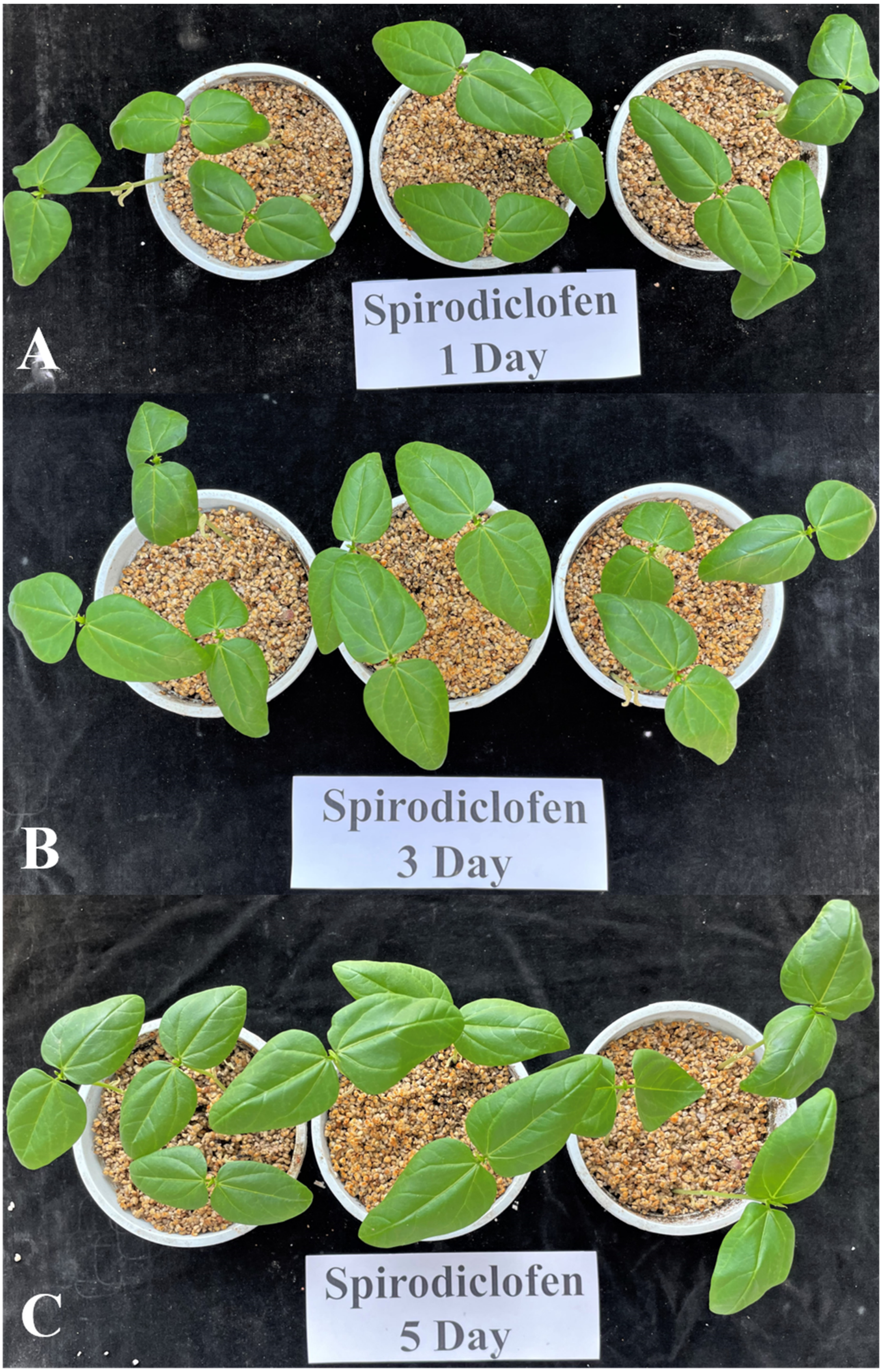

Figure S3. Pictures of control efficiency of piperine (**1**) after 1st day (A), 3rd day (B), and 5th day (C) against *T. cinnabarinus* in the greenhouse.

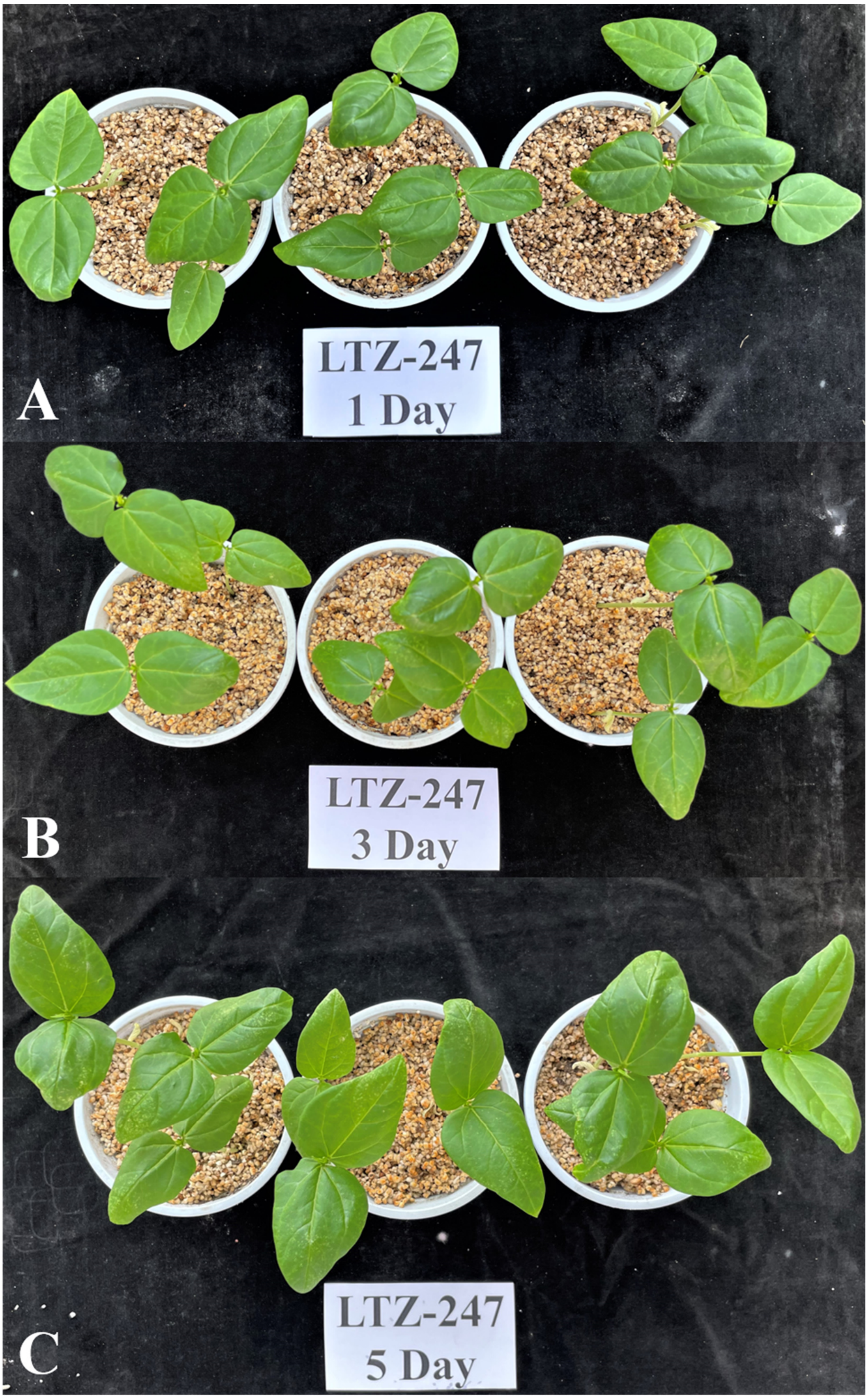

Figure S4. Pictures of control efficiency of compound **9** (LTZ-247) after 1st day (A), 3rd day (B), and 5th day (C) against *T. cinnabarinus* in the greenhouse.

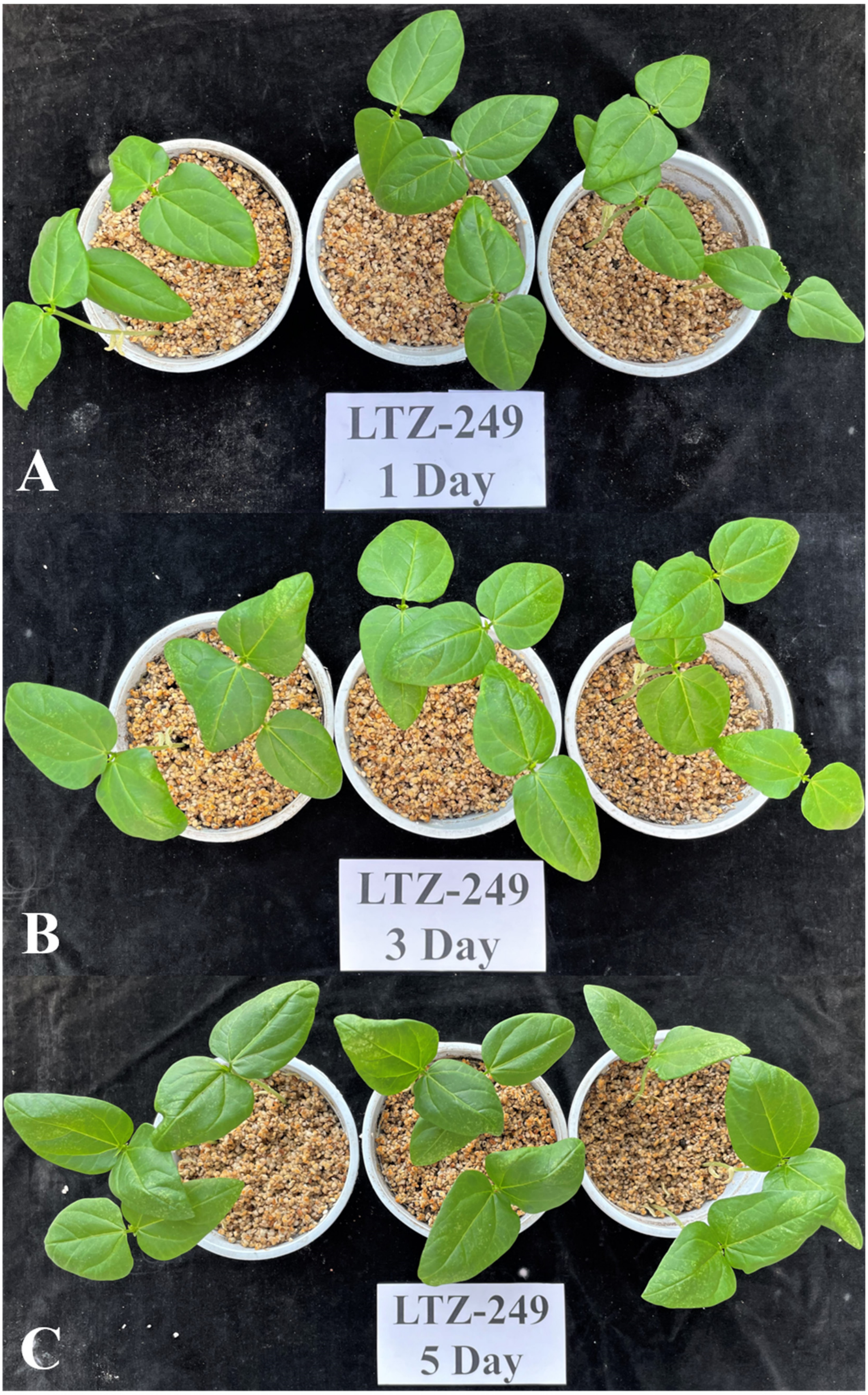

Figure S5. Pictures of control efficiency of compound **11** (LTZ-249) after 1st day (A), 3rd day (B), and 5th day (C) against *T. cinnabarinus* in the greenhouse.
